# Specialized Large Language Model Outperforms Neurologists at Complex Diagnosis in Blinded Case-Based Evaluation

**DOI:** 10.3390/brainsci15040347

**Published:** 2025-03-27

**Authors:** Sami Barrit, Nathan Torcida, Aurelien Mazeraud, Sebastien Boulogne, Jeanne Benoit, Timothée Carette, Thibault Carron, Bertil Delsaut, Eva Diab, Hugo Kermorvant, Adil Maarouf, Sofia Maldonado Slootjes, Sylvain Redon, Alexis Robin, Sofiene Hadidane, Vincent Harlay, Vito Tota, Tanguy Madec, Alexandre Niset, Mejdeddine Al Barajraji, Joseph R. Madsen, Salim El Hadwe, Nicolas Massager, Stanislas Lagarde, Romain Carron

**Affiliations:** 1 Neurosurgery, Université Libre de Bruxelles, 1070 Brussels, Belgium; se471@cam.ac.uk (S.E.H.); nmassage@chu-tivoli.be (N.M.); 2 Neurosurgery, CHU Tivoli, 7110 La Louvière, Belgium; 3 Neurodynamics Laboratory, Department of Neurosurgery, Boston Children’s Hospital, Harvard Medical School, Boston, MA 02115, USA; joseph.madsen@childrens.harvard.edu; 4 Sciense, New York, NY 10013, USAalexandre.niset@gmail.com (A.N.); mejdi.albarajraji@gmail.com (M.A.B.); romain.carron@ap-hm.fr (R.C.); 5 Neurology, Université Libre de Bruxelles, 1050 Brussels, Belgium; bertil.delsaut@gmail.com; 6 Anesthésie-Réanimation, GHU Paris, Pôle Neuro, 75014 Paris, France; aurelien.mazeraud@gmail.com; 7 Neurosciences, Université de Paris, 75006 Paris, France; 8 Neurophysiology and Epileptology, Universite de Lyon, 69007 Lyon, France; sebastien.boulogne@chu-lyon.fr; 9 Neurology, CHU de Nice, Université Côte d’Azur, UMR2CA, 06000 Nice, France; benoit.j@chu-nice.fr; 10 Neurology, Université Catholique de Louvain, Clinique Saint-Pierre Ottignies, 1348 Louvain-la-Neuve, Belgium; timothee.carette@gmail.com; 11 LIP6, CNRS, Sorbonne Université, 75005 Paris, France; thibault.carron@gmail.com; 12 Neurology, CHU Tivoli, 7110 La Louvière, Belgium; 13 Clinical Neurophysiology, CHU Amiens Picardie, CHIMERE UR 7516 UPJV, 80054 Amiens, France; diab.eva@chu-amiens.fr; 14 Neurophy Lab, Université Libre de Bruxelles, 1050 Brussels, Belgium; hugokermorvant@gmail.com; 15 Neurology, La Timone Hospital, AP-HM, 13385 Marseille, France; adil.maarouf@ap-hm.fr; 16 Department of Neurology, Maladie Inflammatoire du Cerveau et de la Moelle Epinière (MICeME), Aix Marseille Université (AMU), CNRS, CRMBM, 13385 Marseille, France; 17 Department of Neurology, Universitair Ziekenhuis Brussel (UZ Brussel), 1090 Brussels, Belgium; 18 NEUR Research Group, Vrije Universiteit Brussel (VUB), 1090 Brussels, Belgium; 19 Evaluation and Treatment of Pain, FHU INOVPAIN, La Timone Hospital, AP-HM, 13385 Marseille, France; sylvain.redon@ap-hm.fr; 20 Neurology, CHU Grenoble, 38700 Grenoble, France; 21 Cabinets de Neurologie d’Allauch et Plan de Cuques, 13190 Allauch, France; sofiene.hadidane@gmail.com; 22 Neuro-Oncology, AMU, La Timone Hospital, AP-HM, 13005 Marseille, France; vincent.harlay@ap-hm.fr; 23 Neurology, CHU Helora, 7000 Mons, Belgium; 24 Neurology, Hospital of Noumea, 98800 Nouméa, France; tanguy.madec.raybaud@gmail.com; 25 Emergency Medicine, Université Catholique de Louvain, 1348 Louvain-la-Neuve, Belgium; 26 Pediatric Intensive Care Unit, Cliniques Universitaires Saint-Luc, 1200 Brussels, Belgium; 27 Département des Neurosciences Cliniques, Centre Hospitalier Universitaire Vaudois (CHUV), 1005 Lausanne, Switzerland; 28 Clinical Neuroscience, University of Cambridge, Cambridge CB2 1TN, UK; 29 AMU, INSERM, Institut Neuroscience des Systèmes (INS), 13005 Marseille, France; stanislas.lagarde@gmail.com; 30 APHM, Timone Hospital, Epileptology and Cerebral Rhythmology, 13005 Marseille, France; 31 Stereotactic and Functional Neurosurgery, La Timone Hospital, AP-HM, 13385 Marseille, France

**Keywords:** artificial intelligence, large language models, neurological diagnosis, clinical decision support

## Abstract

**Background/Objectives**: Artificial intelligence (AI), particularly large language models (LLMs), has demonstrated versatility in various applications but faces challenges in specialized domains like neurology. This study evaluates a specialized LLM’s capability and trustworthiness in complex neurological diagnosis, comparing its performance to neurologists in simulated clinical settings. **Methods**: We deployed GPT-4 Turbo (OpenAI, San Francisco, CA, US) through Neura (Sciense, New York, NY, US), an AI infrastructure with a dual-database architecture integrating “long-term memory” and “short-term memory” components on a curated neurological corpus. Five representative clinical scenarios were presented to 13 neurologists and the AI system. Participants formulated differential diagnoses based on initial presentations, followed by definitive diagnoses after receiving conclusive clinical information. Two senior academic neurologists blindly evaluated all responses, while an independent investigator assessed the verifiability of AI-generated information. **Results**: AI achieved a significantly higher normalized score (86.17%) compared to neurologists (55.11%, *p* < 0.001). For differential diagnosis questions, AI scored 85% versus 46.15% for neurologists, and for final diagnosis, 88.24% versus 70.93%. AI obtained 15 maximum scores in its 20 evaluations and responded in under 30 s compared to neurologists’ average of 9 min. All AI-provided references were classified as relevant with no hallucinatory content detected. **Conclusions**: A specialized LLM demonstrated superior diagnostic performance compared to practicing neurologists across complex clinical challenges. This indicates that appropriately harnessed LLMs with curated knowledge bases can achieve domain-specific relevance in complex clinical disciplines, suggesting potential for AI as a time-efficient asset in clinical practice.

## 1. Introduction

Artificial intelligence (AI) has become an instrumental force across multiple sectors, notably in healthcare [1] and biomedical research [2]. Within this expansive realm, large language models (LLMs) have garnered attention for their proficiencies in natural language processing (NLP). These models have demonstrated versatility in diverse, broad applications, most recently exemplified by the prominent advent of conversational agents [3,4,5]. However, their deployment in specialized scientific domains, particularly medicine, is distinctly challenging [6] due to the stringent constraints inherent to medical applications and the nuanced, discipline-specific considerations such domains entail [7]. Neurology—with its intricate clinical manifestations, neural substrates, and interdisciplinary integration—is a prime example of a complex and rapidly evolving expanse of knowledge that may be substantially embedded—and effectively encoded—in natural language. In practice, neurologists skillfully elicit detailed physical examination findings, which they then integrate with data from diverse diagnostic modalities through sophisticated clinical reasoning pathways defining high-stakes medical management.

Hence, a salient challenge resides in fine-tuning LLMs to achieve domain-specific relevance. Traditional fine-tuning methods are resource-intensive, requiring substantial computational and human capital [8]. Consequently, these methods are often feasible only for large-scale projects with considerable resources [9]. Another limitation of conventional LLM implementation is interpretability and transparency in information processing [10]—a critical requirement for verifiable information generation for medical and research purposes. Furthermore, while modern LLMs have expanded context windows, they still face attention degradation across long contexts, potentially limiting their effectiveness in complex, data-rich environments typical of healthcare and research [11,12]. Here, we evaluate a specialized LLM’s capability and trustworthiness in complex neurological diagnosis, comparing its performance to neurologists in simulated clinical settings.

## 2. Materials and Methods

### 2.1. AI System

**Figure 1:**
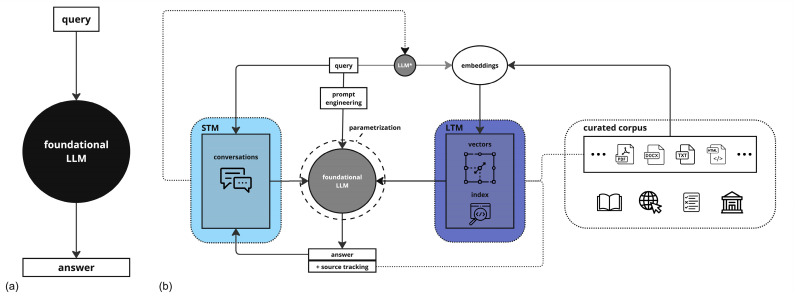
(**a**) Black-box use of LLM. (**b**) AI solution’s architecture. LLM: large language model; * fine-tuned LLM for embedding generation; LTM: long-term memory; STM: short-term memory.

Neura (Sciense, New York, NY, USA) is a solution deploying an LLM with custom parameters and prompt engineering on curated corpora with extended contexts for advanced grounding through retrieval-augmented generation (RAG) [13]. This solution is predicated on a dual-database architecture integrating both ‘long-term memory’ (LTM) and ‘short-term memory’ (STM) components. The LTM serves as the repository for domain-specific knowledge. It employs an agnostic, vectorized approach enabled by text embeddings generated from parsed source texts [14]. The STM captures the setting and conversational history between the user and the LLM, thereby adding a layer of contextual knowledge. The STM is implemented using a non-relational database [15], ensuring real-time accessibility and state persistence of conversational data. Information retrieval is optimized in speed and accuracy through a single-stage filtering process, integrating vector and metadata indexes into a unified structure [16] that integrates vector and metadata indexes into a unified structure, enabling simultaneous semantic similarity matching and exact term identification. This approach reduces computational overhead while maintaining context sensitivity This dual-database architecture with LTM vectorization and STM context capture aims to address attention degradation challenges in long clinical contexts. Source tracking is enabled, culminating in actionable, standardized references for the end-user and ensuring verifiability of answer accuracy. For this study, we deployed a state-of-the-art LLM, GPT-4 Turbo (OpenAI, San Francisco, CA, USA) [5], on a prototype corpus curated for clinical neurology sourced from five comprehensive neurology textbooks [17,18,19,20,21], the neurologic disorders section of Merck’s Manual (copyrighted) [22], and Wikipedia (open-source) [23] (Figure 1).

### 2.2. Diagnostic Challenges

**Figure 2:**
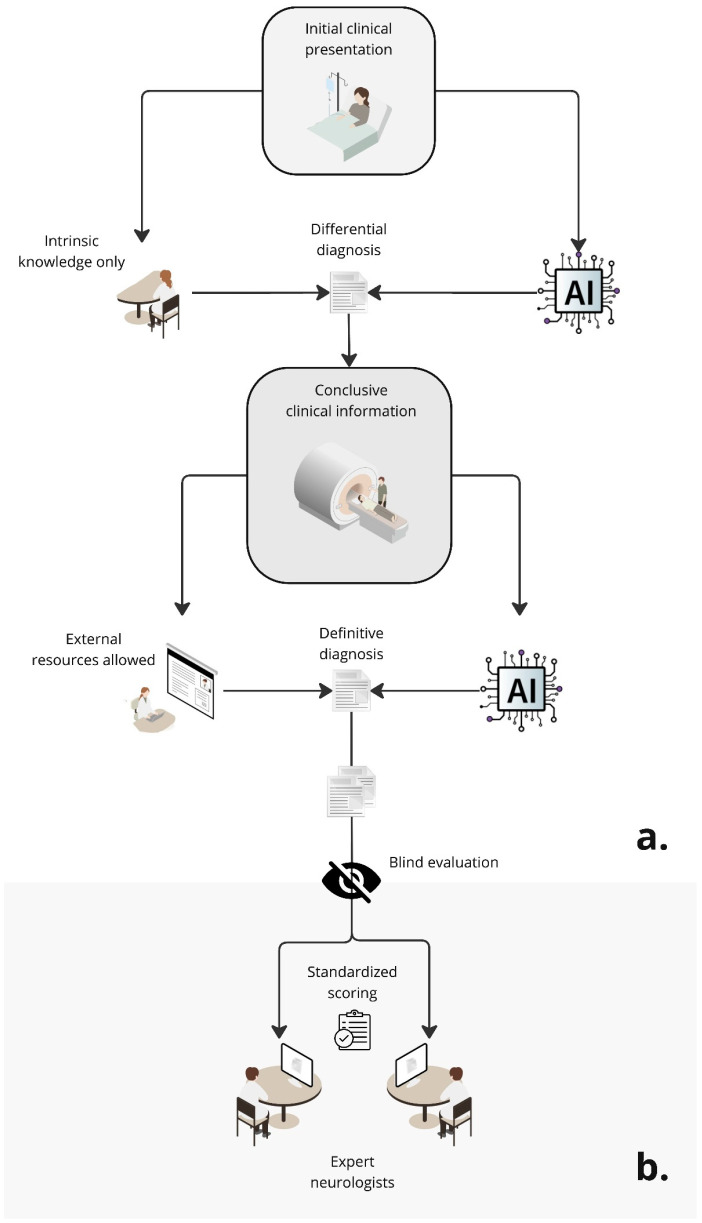
Overview of the simulated clinical diagnostic challenge: a videoconference setup for clinical scenarios simulations with human neurologists and specialized AI through grounding, parametrization, and prompt engineering; (**a**) shows the two-tiered diagnostic workflow from initial clinical presentation to differential diagnosis (using intrinsic knowledge only for human neurologists), followed by conclusive clinical information leading to definitive diagnosis (with external resources allowed for human neurologists); (**b**) represents the blind evaluation process where expert neurologists performed standardized scoring of anonymized responses from both human participants and AI.

Five representative clinical scenarios were adapted from peer-reviewed complex cases [24,25,26,27,28] to mirror the clinical practice through a two-tiered diagnostic approach. These cases were selected to represent diverse neurological subspecialties and encompass a spectrum of diagnostic complexity requiring the integration of clinical and paraclinical findings (Table 1). The first tier required formulating and justifying an exhaustive differential diagnosis based on initial clinical presentation and findings. In the second tier, conclusive clinical information was provided to establish a definitive diagnosis (Table 2). We recruited senior residents and board-certified neurologists from teaching hospitals. Neurologists engaged in complex clinical reasoning to solve these diagnostic challenges, solely relying on intrinsic knowledge in the first tier—external resources were permitted in the second tier. All challenges were conducted via videoconferencing sessions, supervised by two investigators who provided documents presenting the cases (Supplementary S1), initial instructions, and procedural assistance. Answers with timing were recorded in text documents, which were subsequently collected and anonymized. AI undertook the challenges based on the same documents provided to neurologists. Answers were time-stamped and anonymized. Two senior academic neurologists, each responsible for residency training and educational programs at their respective universities, independently evaluated the answers, blinded to the involvement of AI as a participant. They employed a standardized scoring sheet (Supplementary S2), assigning points for precise and justified diagnoses and allowing bonus points for unexpected, relevant findings (Figure 2).

Incorrect or risky conclusions incurred deductions, with a two-point loss yielding a null question score. Null scores from both evaluators constituted question failure. If multiple participants achieved the maximum score for a given question, the evaluator chose a preferred answer; conversely, if a single answer attained the maximum score, it was then defined as the highest score. In parallel, an independent investigator assessed the verifiability and reliability of the AI-generated information. This was achieved by classifying the references provided within the answers as relevant, irrelevant, or hallucinatory (i.e., incorrect or nonexistent) (Supplementary S3).

### 2.3. Statistical Analysis

Descriptive statistics were calculated for the scores and times. For normalization, the maximum possible combined score for each question was determined by summing the highest score assigned by each evaluator. For any participant, we calculated the combined score from both evaluators for each question and then divided this by its maximum possible combined score. These resulting normalized scores were expressed as percentages, indicating the proportion of the maximum possible points each participant collected on a given question. We used the intraclass correlation coefficient (ICC) to measure consistency agreement for inter-rater reliability between evaluators. We compared the performance of the AI with that of neurologists using a linear mixed-effects model. Before analysis, we used residual plots, QQ plots, and Shapiro–Wilk tests to assess the assumptions of normality, homoscedasticity, and random effect structure. This model utilized average scores derived from the two evaluators as the dependent variable. Participant type (AI vs. human) was treated as a fixed effect, while variability across questions was modeled as random. The significance of the fixed effect was corroborated using an ANOVA with Satterthwaite’s method for approximating degrees of freedom. We employed a Monte Carlo simulation (MCS) of 10,000 iterations to estimate the probabilities for AI achieving observed thresholds of maximum scores, highest scores, and preferred answers among its 20 scores by chance—assuming a uniform distribution of scores within each question’s specific range across all participants. We set our alpha level threshold at 0.05 to determine statistical significance using two-tailed tests. All computations and visualizations were performed using R version 4.1.3, with the packages ‘afex’, ‘eulerr’, ‘ggplot2’, ‘irr’, ‘lme4’, and ‘lmertest’.

## 3. Results

**Figure 3:**
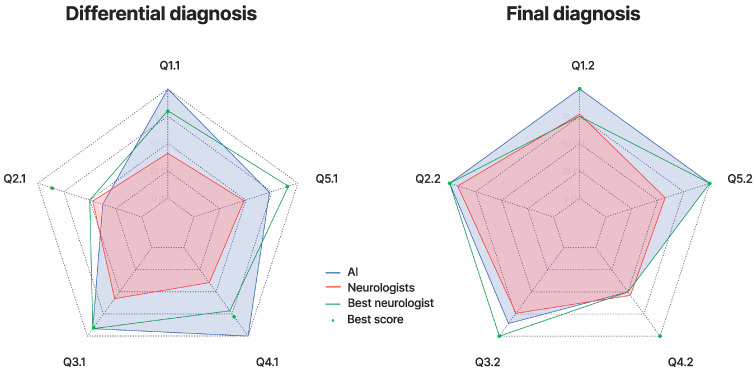
Radar charts of the performance across the differential diagnosis and final diagnosis challenges for AI, all neurologists collectively (‘Neurologists’), the best-performing individual neurologist (‘Best neurologist’), and the best individual score from all neurologists for each question (‘Best score’). Each axis corresponds to a specific question, designated as Qx.y (where ‘x’ is the case number and ‘y’ is the tier), with scores normalized and depicted in increments of 25%.

Of the 13 neurologists, 8 were board-certified. Challenges were conducted between March and October 2023. ICC(C,2) was found to be significant at 0.767 (95% CI [0.675, 0.833], F(139,139) = 4.3, *p*-value < 0.001). The residuals did not significantly deviate from normality (W = 0.99327, *p* = 0.753, Shapiro–Wilk test) as observed on the QQ plot, and plots of residuals versus fitted values supported homoscedasticity (Supplementary Figures S1 and S2). Additionally, random effects for participants and questions showed substantial variance (0.3789 and 0.3587, respectively, with a residual variance of 1.0584). Across all questions, AI achieved a significantly higher normalized score of 86.17% versus 55.11% for neurologists (SD = 14.81, range = 30.85–80.85; averages of 66.38% for residents and 48.07% for board-certified physicians—estimate = 1.46, Std. Error = 0.39, df = 129, t = 3.75, *p* < 0.001, linear mixed-effects model, and F(1,129) = 14.021, *p* < 0.001, type III ANOVA). For differential diagnosis questions, AI achieved a normalized score of 85% versus 46.15% for neurologists (SD = 15.24, range = 26.67–78.33; averages of 58.45% for residents and 39.40% for board-certified physicians). For final diagnosis, AI achieved a normalized score of 88.24% versus 70.93% for neurologists (SD = 17.36, range = 35.29–97.06; averages of 80.5% for residents and 64.87% for board-certified physicians) (Figure 3). AI performance notably decreased for differential diagnosis in Q2.1 and final diagnosis in Q4.2. In Q2.1, AI proposed broad diagnoses, including neoplastic and leukodystrophic conditions that evaluators deemed aberrant. In Q4.2, AI initially proposed neurosarcoidosis in the differential diagnosis but ultimately favored paraneoplastic neuropathy, a choice similarly made by most neurologists; this misalignment resulted in a null score due to the conservative scoring approach of one evaluator.

**Figure 4:**
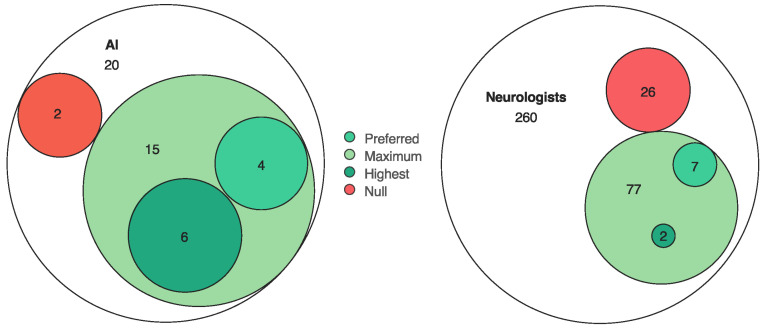
Euler diagrams representing AI and neurologists’ answer attributes for AI, showing total and respective counts and proportions of evaluations in each category—null scores, maximum scores, highest scores, and preferred answers.

The mean number of null scores and question failures was 2 and 0 for AI and 2 and 0.46 for neurologists (1 and 0.2 for residents and 2.625 and 0.625 for board-certified physicians). AI obtained 15 maximum scores (*p*-value < 0.001, MCS) in its 20 evaluations, 6 of the 8 highest scores (*p*-value < 0.001, MCS), and 4 of the 11 preferred answers from both evaluators (*p*-value = 0.03, MCS) (Figure 4).

**Figure 5:**
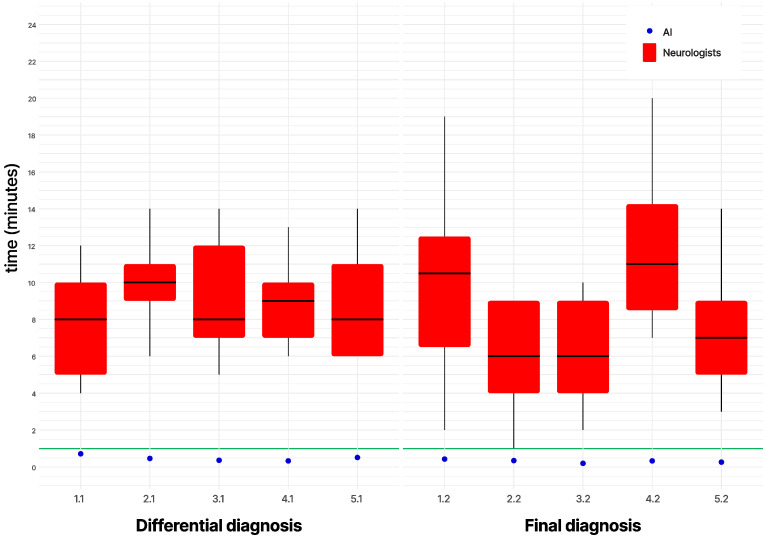
Box plots displaying the distribution of neurologists’ response times, with AI’s times as distinct points—horizontal green lines mark a one-minute reference.

In comparison, the best neurologist, a resident, obtained a normalized score of 80.85%, with nine maximum scores, including two highest scores from one evaluator without a preferred answer and one null score. Neurologists’ mean response times for differential and final diagnosis were 9.62 (SD = 4.47, range = 4–32) and 8.85 min (SD = 5.53, range = 1–30), compared to AI’s mean times of 28.8 and 19.2 s, respectively (Figure 5).

All references provided by the AI were classified as relevant, and the generated information was deemed accurately derived from the cited sources. Despite the diverse corpus including Wikipedia and Merck’s Manual, source tracking revealed the AI system exclusively retrieved information from the peer-reviewed neurology textbooks for all diagnostic challenges. No instances of hallucinatory content were detected.

## 4. Discussion

In this blinded, controlled, comparative study, AI demonstrated superior diagnostic performance compared with a cohort of 13 neurologists across five complex clinical challenges, as evaluated by two academic neurologists. It achieved a significantly higher normalized score of 86.17% against the neurologists’ 55.11%, with 15 maximum scores (including 6 highest scores) out of 20 evaluations. Across various levels of experience (residents and board-certified physicians) and types of diagnosis (differential and final), the AI consistently outperformed the neurologists. Interestingly, senior residents outperformed board-certified neurologists, a trend that might be attributed to the broader, ongoing training of the former, contrasting with the deep but narrow specialization of the latter. As this may reflect a judicious case selection for representing general neurology, it also raises the question of artificial and human acumen in niche or emerging domains of expertise.

In the diagnostic challenges, each tier encapsulated distinct aspects of clinical practice: the first tier encompassed bedside diagnosis, utilizing intrinsic knowledge and initial clinical presentation, while the second tier, extending to broader clinical investigation, allowed the inclusion of external resources for definitive diagnosis. Consequently, the gap between neurologists’ normalized scores for differential (46.15%) and final diagnosis (70.93%) indicates that external resources likely enhance diagnostic accuracy beyond mere reliance on personal knowledge. In contrast, AI performed well in both differential (85%) and final diagnosis (88.24%), demonstrating capability in two distinct yet complementary diagnostic tasks: the substantiated formulation of a comprehensive array of hypotheses and the decisive synthesis of a conclusive diagnosis. Moreover, AI manifested perspicuity and cogency, with a significant record of four preferred answers selected by both evaluators. Nonetheless, neurology is a discipline reliant on collaborative, multidisciplinary problem-solving. In turn, comparing AI with neurologists’ highest individual scores reveals a nuanced picture. In differential diagnosis, both the best neurologists and AI surpassed each other on two occasions. Remarkably, at least one neurologist achieved perfect scores for all final diagnoses, surpassing AI in two cases. Of course, using the highest individual scores as a proxy for collective intelligence does not fully capture the complex dynamics that influence the collective endorsement of individual contributions. In addition, AI’s rapid generation of differential and final diagnoses, typically within one minute, contrasts the average times of 10 and 9 min, respectively, taken by neurologists. This disparity, particularly considering the neurologists’ consistent reliance on external resources for final diagnoses, underscores the potential of AI as a time-efficient and resourceful asset in clinical practice, especially for individual clinical endeavors. It suggests workflow optimization potential, allowing physicians to focus more on patient interaction and treatment planning, though implementation frameworks must ensure AI remains a complementary tool that enhances rather than replaces clinical judgment.

Regarding AI’s null scores, the first evaluator incurred a two-point deduction on a differential diagnosis challenge because the two least important diagnoses proposed were deemed aberrant (Supplementary S4). Otherwise, AI’s answer would have received a score of 4 (out of 5 possible points based on this evaluator’s scoring pattern for this question). Notably, the answer included the correct final diagnosis. This outcome likely resulted from the AI’s prompt engineering strategy, inspired by ‘surgical sieves’ [29] to systematically evaluate various pathologies, leading to the listing of supernumerary diagnoses. Paradoxically, this reveals that while this algorithmic design enables the LLM to emulate a human approach to diagnosis, it lacks innate human nuance in selecting relevant diagnoses—an intuition not emerging from our solution architecture and LLM’s foundational weights. Context-sensitive filtering and adaptive mechanisms of prompt engineering could better prioritize diagnoses based on clinical relevance while maintaining comprehensive differential coverage—addressing the gap between algorithmic thoroughness and human clinical intuition in diagnostic decision-making. The second evaluator attributed a null score on a final diagnosis challenge, applying a deliberately conservative scoring approach—i.e., recognizing only a unique option as granting points (Supplementary S4). While AI initially proposed the correct diagnosis in its differential, it did not retain it in its final diagnosis. Interestingly, the AI’s final diagnosis aligned with most neurologists (11 out of 13 human participants), leading to the highest number of null scores on a question record—questioning AI’s ability for original contributions.

### 4.1. Rationale

Since the public introduction of LLMs, many studies have compared AI and human performance. As this surge emphasizes the potential of generalist models, broadening accessibility for users of varying expertise, it also prompts scrutiny of their reliability in specialized contexts [30,31]. Indeed, these models were often applied to large datasets and within paradigms distant from real-world intricacies. On the other hand, seminal works have demonstrated promising results from large-scale projects conducted by major technology corporations, employing resource-intensive fine-tuning methods in sophisticated protocols [9,32]. However, many of these studies yielded outcomes that proved difficult to interpret [33]. We posited that a state-of-the-art foundational LLM, adeptly harnessed to a curated and indexed corpus of knowledge, can achieve domain-specific relevance in a complex clinical discipline. In parallel, we pursued controllability, verifiability, and scalability for mitigating influences and biases from the model’s pre-trained weights, enabling source tracking and advancing accessibility for clinicians and researchers. Testing this hypothesis, we conducted an in-depth, qualitative, and quantitative comparison of human and artificial diagnostic acumen, perspicuity, and cogency in a naturalistic setting. The findings support our rationale by confirming effective information synthesis from relevant selections of aptly cited sources and asserting the absence of hallucinatory content. In addition, this solution aims for versatility and accessibility. Relying on open-source technologies, it can be implemented on multiple foundational LLMs (e.g., GPT [34], Llama [35], and the Mistral series [36]). Its data-agnostic architecture accommodates numerous file formats for building a curated corpus.

### 4.2. Limitations

The limited cohort of neurologists and the select set of clinical cases do not fully represent the spectrum of neurological expertise or the complexities of clinical practice, potentially limiting the generalizability of the findings and constraining the solution’s broader applicability. Regarding the reliability of the AI-generated information, our two-fold approach—first categorizing the relevance of provided references and then applying binary classification of information accuracy—does not capture the subtle influences of the LLM’s constitutional weights. Indeed, the foundational LLM integral to our framework is inherently subject to biases [37,38], a byproduct of their data-driven training that lies beyond our control. This issue is exacerbated by proprietary and closed-source models [39] which also exposed to overfitting. Biases do not spare the corpus we employ [40], despite—and, in some aspects, because of—our control over its curation. Interestingly, despite access to diverse sources, the system’s exclusive retrieval from peer-reviewed textbooks suggests an inherent selection mechanism favoring authoritative academic content. However, even rigorously vetted academic sources harbor inherent biases, emphasizing the critical importance of transparent source attribution that enables users to evaluate information provenance. This point and the curation and maintenance of the corpus itself were beyond the scope of this study. It raises intellectual property considerations and concerns about the quality and up-to-dateness of the information. As this research is nascent, we focused on solution development rather than its optimization. The system’s performance can vary based on several factors, including the fine-tuned LLM for embeddings, the foundational LLM, their parameters, the prompt engineering strategies, and the structure and composition of the LTM and STM databases. The prototype corpus was assembled from raw, unprocessed textual data to uphold methodological neutrality and facilitate the agnostic, versatile approach we aimed to investigate. It remains plausible that text preprocessing techniques optimized for LLMs/NLP could enhance the system’s performance. Also, we purposely deployed our solution on a limited but cohesive corpus of knowledge on clinical neurology. Further investigation is required to assess the solution’s ability to manage a diverse and extensive corpus, including heterogeneous and conflicting sources, in a manner that effectively and transparently meets the end-user’s needs and objectives. Recently, multimodal AI models [41,42], which can integrate natural language information with other sensory data such as images and audio, were introduced. This framework is not yet equipped to deploy these models. Finally, evaluating the solution’s usability for non-expert users was beyond the scope of this study and will be the focus of subsequent research.

### 4.3. Perspectives

Humans have built, shared, and accessed knowledge in evolving ways. Transitioning from orality to literacy, and from analog to digital media, these evolutions have fundamentally shaped our comprehension of the world. Throughout these transitions, natural language remains central. Its adaptive and symbolic nature enables abstract thought and complex communication. With AI, the human ability for information integration and generation has been challenged—LLMs’ prowess for NLP is humbling in this regard. It elucidates the singular role of natural language in structuring knowledge derived from various modalities, media, and agents. This prompts reconsideration of complex tasks once thought uniquely human. While LLMs excel at information-intensive tasks, they lack general reasoning capabilities [43,44,45,46,47] and are grounded in human-derived data. This raises questions about their efficacy in scenarios requiring original thinking or high-order cognition and about potential bias propagation [48].

LLMs indicate a new phase of human–machine integrative intelligence, with profound implications for cognition and knowledge. In medicine, LLMs can complement clinicians’ experiential understanding by integrating patient histories, physical examinations, and test findings with vast medical knowledge. This emphasizes the need for human oversight and contextual interpretation.

**Figure 6:**
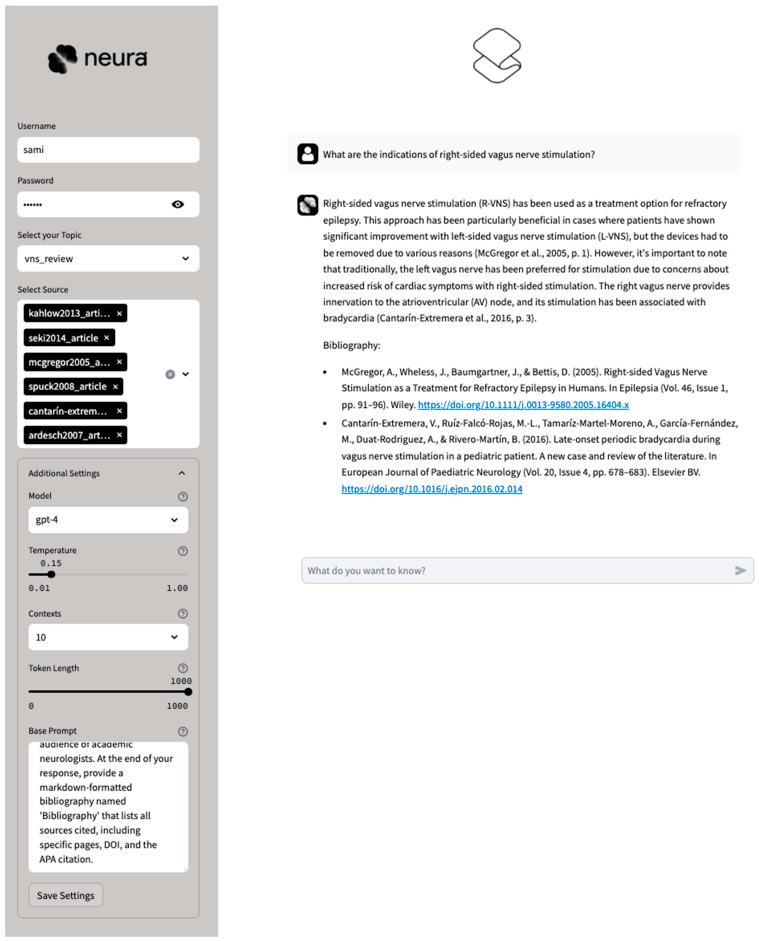
Web purpose-built interface in basic mode provides the ability to select a corpus, define a knowledge base with specific sources, choose the foundational LLM deployed, parameterize its behavior, and set base prompt engineering.

Explainable AI (XAI) and specialized, purpose-built interfaces (Figure 6) will be crucial for ethical integration and utility evaluation in high-stakes fields. It is imperative to involve and empower clinicians and scientists in LLM-driven applications and research, developing accessible tools and consensus-based standards aligned with medical needs and priorities. This approach ensures that as our tools become more sophisticated, they remain controllable and serve our purposes rather than obfuscate them.

## 5. Conclusions

In our comparative analysis, a specialized LLM demonstrated superior diagnostic performance against practicing neurologists in complex neurological cases, highlighting its potential as a time-efficient clinical asset. The solution’s architecture employing RAG on a curated corpus achieved domain-specific relevance while maintaining verifiability through source tracking. Our findings suggest a promising trajectory for human–machine integrative intelligence in healthcare. This research underscores the importance of developing accessible, transparent AI tools that complement rather than replace clinical expertise. As we advance these technologies, maintaining human oversight and aligning development with medical priorities will ensure AI systems remain valuable tools that enhance rather than diminish the essential human dimensions of healthcare.

## Figures and Tables

**Table 1:** Concise summaries of clinical presentations, neurologic fields, and final diagnoses for illustrative neurology cases demonstrating diagnostic challenges across diverse neurological disorders.

	Case Summary	Neurological Field	Final Diagnosis
Case 1	An 84-year-old Chinese woman with recurrent focal deficits, cognitive decline, multifocal cerebral artery constriction, and bilateral infarctions.	Neurovascular/neuro-oncology	Intravascular lymphoma
Case 2	A 44-year-old woman with hypothyroidism presenting with cognitive decline, headaches, confusion, and progressive multifocal white matter lesions.	CNS inflammation diseases	Susac disease
Case 3	A 50-year-old woman with systemic lupus erythematosus presenting with acute progressive left-sided weakness and sensory deficits, with imaging showing rapidly worsening right-hemispheric lesions.	Demyelinating diseases	Neuromyelitis optica spectrum disorder (NMOSD)
Case 4	A 65-year-old man with diabetes presenting with progressive asymmetric weakness, sensory deficits, areflexia, and weight loss.	PNS inflammation diseases	Neurosarcoidosis
Case 5	A 55-year-old man with extensive psychiatric history presenting with subacute-on-chronic cognitive decline, dysphagia, involuntary hyperkinetic movements, gait instability, and chronic transaminitis.	Movement disorders	Neuroacanthocytosis

**Table 2:** Illustrative example of diagnostic workflow and scoring (Case 5). Structured clinical scenario highlighting the diagnostic reasoning process, scoring criteria for differential diagnoses (tier 1) and the final diagnosis (tier 2), demonstrating the study methodology.

Initial Presentation	Differential Diagnosis (Tier 1)	Ancillary Exams and Results	Final Diagnosis (Tier 2)
- Hyperkinetic involuntary movements affecting trunk and limbs, inability to suppress movements; - Chronic psychiatric conditions (PTSD, schizophrenia, anxiety, depression); - Chronic dysphagia, regurgitation, elevated transaminases (AST: 304 U/L, ALT: 154 U/L); - CK elevation (4753 IU/L, baseline ~600–800 IU/L); - MRI: bilateral caudate atrophy.	Evaluators awarded points (max 1 each) for justified differential diagnoses: - Basal ganglia structural disorders (vascular, demyelinating); - Toxic-metabolic disorders (electrolyte derangements, hyperglycemia); - Drug-induced (antipsychotics, tardive dyskinesia); - Systemic autoimmune (lupus, APLS); - Hereditary (Huntington’s, neuroacanthocytosis, Wilson); - Paraneoplastic (anti-CRMP5/NMDA). Penalties for aberrant/harmful hypotheses (max − 2). Bonuses for plausible, justified alternative diagnoses (max + 2).	- Serum copper and ceruloplasmin normal; - Extensive gastroenterologic and rheumatologic evaluations unrevealing; - MRI brain: bilateral caudate atrophy.	Neuroacanthocytosis (3 points awarded). (Alternative, less accurate diagnosis, Huntington’s: 1 point.) Penalties possible for aberrant/harmful conclusions.

## Data Availability

Anonymized data not published within this article will be made available by request to the corresponding author from any qualified investigator upon reasonable request.

## Supplementary Materials

The following supporting information can be downloaded at: https://www.mdpi.com/article/10.3390/brainsci15040347/s1, Supplementary S1. Clinical cases; Supplementary S2. Scoring sheets; Supplementary S3. AI Sourced Replies; Supplementary S4. AI’s null scores; Figure S1: Normal Q-Q plot; Figure S2: Plot of residuals. References [49,50,51,52,53] are cited in the supplementary materials.

## Abbreviations

The following abbreviations are used in this manuscript:

AI	Artificial intelligence
LLM	Large language model
RAG	Retrieval-augmented generation
