# Supplementary material for: Specialized Large Language Model Outperforms Neurologists at Complex Diagnosis in Blinded Case-Based Evaluation
Source: Brain Sci. 2025 Mar 27;15(4):347. doi: 10.3390/brainsci15040347 (PMC12025783; doi:10.3390/brainsci15040347)
Supplement: Supplementary file 1 [file brainsci-15-00347-s001.zip › brainsci-3540476-supplementary.pdf]

Supplementary Materials

Supplement to: Barrit S et al. *Specialized Large Language Model Outperforms Neurologists at Complex Diagnosis in Blinded Case-based Evaluation*

This material has been provided by the authors to give readers additional information about their work.

Table of contents

Supplementary S1. Clinical cases ***3***

Case 1 **3**

Case 2 **5**

Case 3 **8**

Case 4 **10**

Case 5 **14**

Supplementary S2. Scoring sheets ***16***

Case 1 **16**

Case 2 **17**

Case 3 **18**

Case 4 **19**

Case 5 **20**

Supplementary S3. AI Sourced Replies ***21***

Case 1 - Q1 **21**

Case 1 - Q2 **22**

Case 2 - Q1 **23**

Case 2 - Q2 **25**

Case 3 - Q1 **26**

Case 3 - Q2 **28**

Clinical Presentation: 28

MRI Findings: 28

Laboratory Findings: 28

Response to Treatment: 28

Brain Biopsy: 28

Case 4 - Q1 **28**

Case 4 - Q2 **30**

Case 5 - Q1 **31**

Case 5 - Q2 **32**

Supplementary Figure S1. Normal Q-Q plot ***34***

Supplementary Figure S2. Plot of residuals ***35***

Supplementary S4. AI's null scores ***36***

Case 2 **36**

Case 4 **38**

Supplementary S1. Clinical cases

Case 1

- **Initial presentation**

A healthy 84-year-old woman of Chinese ethnicity presented with multiple separate episodes of focal neurologic deficits (right-sided weakness with aphasia, as well as left-sided weakness) over 2 days, in the context of 3 months of subacute cognitive decline. She had been well leading up to this presentation. CT angiogram showed multifocal constriction of the middle cerebral arteries and bilateral M3 occlusions. Posterior circulation vessels were unremarkable. Cerebral MRI with gadolinium showed multiple foci of restricted diffusion in the bilateral frontal and parietal lobes and diffuse T2/fluid-attenuated inversion recovery changes without abnormal enhancement, consistent with infarction of varying ages (figure, A). She was started on low-dose acetylsalicylic acid and deep vein thrombosis prophylaxis and admitted to the hospital.

  - **What would be your first differential diagnosis for this presentation and explain why (pros and cons)?**

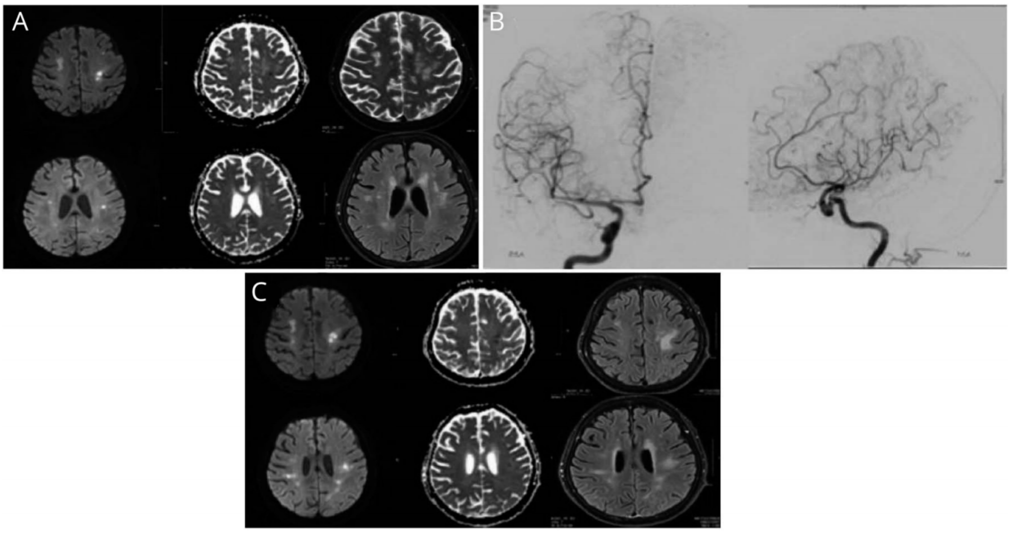

- **Ancillary exams results and follow-up:**

In our patient, a conventional angiogram showed beading of small and medium-sized vessels, both intracranially and extracranially, concerning for a diffuse vasculopathy (figure, B). Bloodwork showed elevated inflammatory markers (erythrocyte sedimentation rate 115, C-reactive protein 56.1), but negative testing for rheumatologic, hypercoagulable, and infectious processes including antiphospholipid antibody syndrome and HIV. CSF analysis demonstrated 2 total nucleated cells, normal glucose of 2.3 mmol/L, elevated protein at 1.46 g/L (normal, 0.18–0.68), and elevated albumin at 0.96 g/L (normal, 0.14–0.25). Identical oligoclonal immunoglobulin G banding was present in both serum and CSF, suggestive of increased permeability of the blood–brain barrier. CSF cytology was negative. CT of the chest, abdomen, and pelvis showed no solid organ malignancy or lymphadenopathy, and paraneoplastic antibodies were negative. Serum and urine electrophoresis were unremarkable. Bilateral temporal artery biopsies demonstrated only nonspecific disruption of internal elastic membranes. The patient was started on high-dose IV solumedrol after consultation with rheumatology. Brain biopsy was deferred after neurosurgical consultation, as the only accessible targets were in proximity to the motor cortex. Despite 2 rounds of high-dose steroids, the patient continued to have recurrent strokes with new ischemic lesions demonstrated on repeat MRI (figure, C). Investigations were continued in parallel due to the lack of diagnostic clarity, including a repeat conventional angiogram and bone marrow biopsy for chronic mild thrombocytopenia and normocytic anemia. Discussions among the patient, family, neurology, and rheumatology teams resulted in a decision to initiate cyclophosphamide. The bone marrow biopsy was completed 2 days after initiation of cyclophosphamide and revealed normocellular marrow with adequate trilineage hematopoiesis with no evidence of lymphoma or other infiltrative pathology. Conventional cerebral angiography demonstrated ongoing beaded appearance of vessels with new involvement of the occipital arteries. Bilateral occipital artery biopsies were taken, which showed nonspecific hyperplasia and loss of internal elastic lamina. The patient's bicytopenia worsened after treatment with cyclophosphamide and Septra, with platelets dropping to 30 × 109/L and hemoglobin dropping to 67 g/L, without signs of bleeding. Septra was stopped, but the bicytopenia worsened. A CT of the chest, abdomen, and pelvis was repeated, demonstrating subtle smooth thickening of the aortic wall (perivisceral segment), extending into the renal arteries bilaterally. IV immunoglobulin was initiated for presumed autoimmune cytopenia and bone marrow biopsy was repeated 3 months after initial presentation. It showed a low level of patchy interstitial marrow infiltration by abnormal large B-lymphocytes, compatible with large B-cell lymphoma, with double expression of C-MYC and BCL2. CD34 stain showed 1 or 2 focal areas of possible intravascular involvement.

  - **What is your final diagnosis and explain why?**

Case 2

- **Initial presentation**

A 44-year-old woman with a history of hypothyroidism taking daily levothyroxine was admitted to an outside hospital with subacute cognitive decline. Her symptoms had started 3 weeks previously with headache, sore neck, and upper respiratory symptoms for which she sought care at a local emergency room. She did not complain of confusion or demonstrate signs of cognitive decline at that time and was discharged home with a prescription for antibiotics. A few days later, she was hospitalized after appearing disoriented, exhibiting signs of confusion, responding slowly, and running into things at work. On examination, she was oriented only to self with poor verbal output and followed only rudimentary commands. She had grasp and left palmomental reflexes. Visual acuity measurement was precluded by her mental status. She blinked to threat and tracked a light with either eye. Upgaze was mildly restricted and she had gaze-evoked nystagmus. Cranial nerve examination was otherwise unrevealing. Muscle tone was normal. All extremities moved spontaneously against gravity. Reflexes were diffusely 3+ with Chaddock signs bilaterally. She responded to stimuli in all extremities. There was no ataxia noted during spontaneous movements. Further testing was limited by her inability to follow commands. Testing performed at the outside hospital included toxicology screen, which was positive for cannabinoids and amphetamines. Blood count and metabolic panel including liver tests were within normal limits. MRI brain demonstrated multiple subcentimeter, nonenhancing T2 hyperintense lesions in supratentorial white matter. CSF analysis demonstrated elevated protein (196 mg/dL) and 5 oligoclonal bands, but normal cell count and glucose. Her mental status spontaneously improved, though not to baseline, and she was discharged with outpatient follow-up. One week later, she was prescribed oral steroids for complaints of headache and hearing loss. Two weeks after her initial discharge, she was readmitted with recurrent and progressive cognitive decline. Repeat MRI demonstrated increased burden of T2 hyperintense lesions in the periventricular white matter and corpus callosum with a lower lesion burden in the subcortical white matter, basal ganglia, and infratentorial structures. None of the lesions demonstrated enhancement. Some of them demonstrated restricted diffusion (figure, A–C). She was transferred to our institution for further evaluation.

  - **What would be your first differential diagnosis for this presentation and explain why (pros and cons)?**

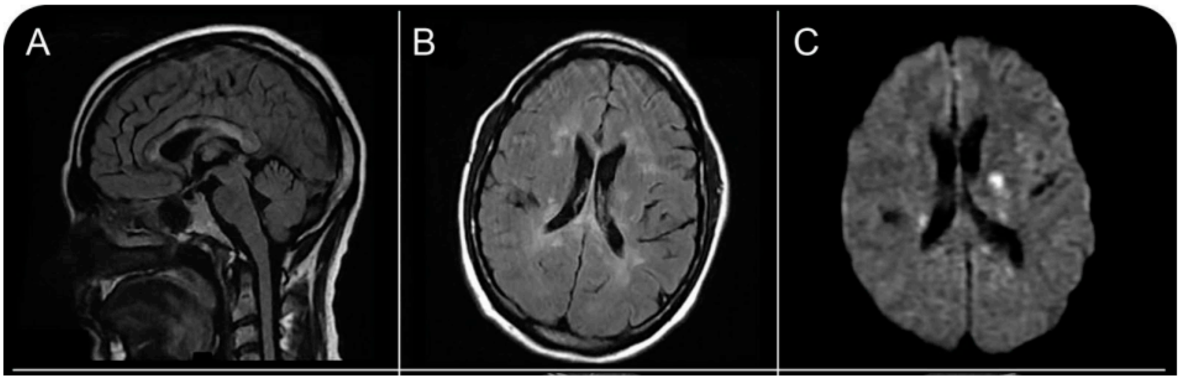

- **Ancillary exams results and follow-up:**

Normal serologic studies included those for antinuclear antibody assay, antineutrophil cytoplasmic antibody, rapid plasma regain, fluorescent treponemal antibody, and anti-Ro/SSA and La/SSB antibodies. Repeat CSF analysis demonstrated 10 leukocytes/mL (100% lymphocytes), no erythrocytes, mildly elevated glucose (83 mg/dL), elevated protein (153 mg/dL), and negative venereal disease research laboratory. Magnetic resonance angiography of the head and neck and 4-vessel cerebral catheter angiography were unrevealing. EEG revealed diffuse slowing without epileptiform abnormalities. Audiometry was precluded by her mental status, but auditory potentials demonstrated no significant abnormalities. Dilated ophthalmoscopic examination was notable for inferotemporal retinal whitening with edema and intraretinal hemorrhage in a perivascular distribution (figure, D), diagnostic of an inferotemporal branch retinal artery occlusion (BRAO). No arteriolar sheathing or Gass plaques were visualized. There was no associated uveitis. Given the dramatic nature of her clinical presentation, a broad workup was performed for treatable subacute dementing illnesses in parallel with the focused evaluation described above. This included evaluation for vitamin deficiency, antiNMDA encephalitis, Hashimoto encephalitis, Wilson disease, infectious encephalitis, neurosarcoidosis, and thromboembolic disease, which were all negative.

  - **What is your final diagnosis and explain why?**

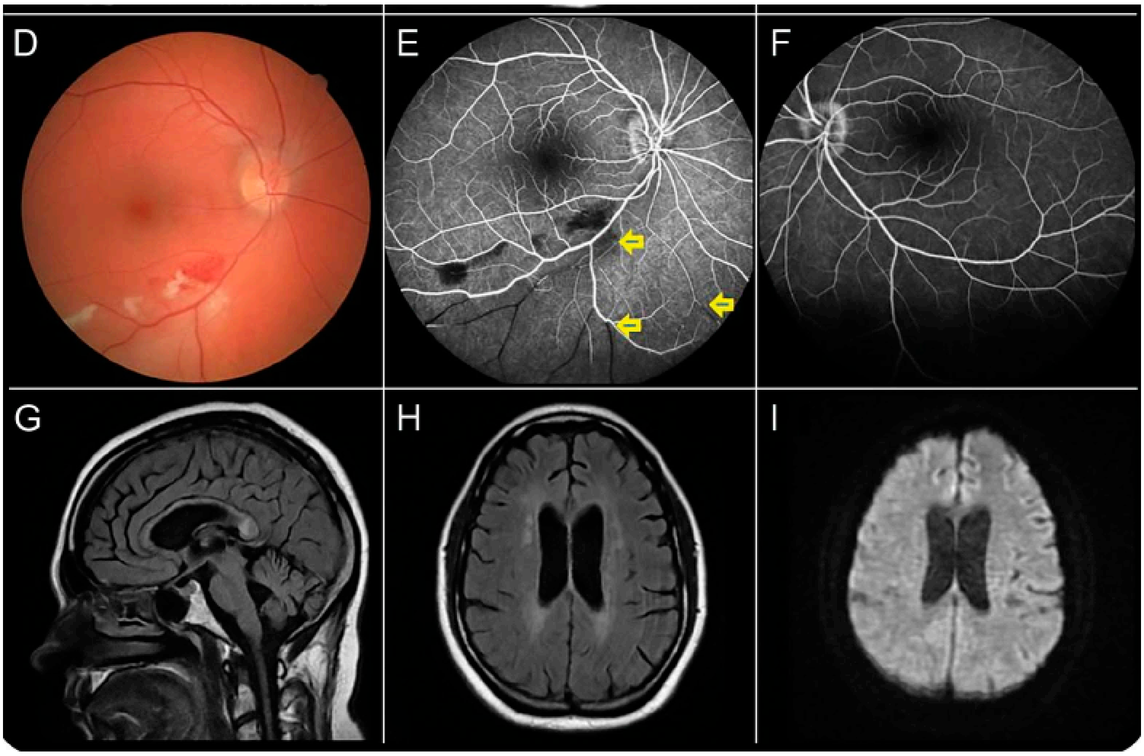
D
E
F
G
H
I

Case 3

- **Initial presentation**

A 50-year-old woman with a history of systemic lupus erythematosus (SLE) treated with methotrexate and hydroxychloroquine presented to the emergency department for evaluation of numbness and weakness of the left hand that began 12 hours earlier. Twenty years ago, the patient had been diagnosed with SLE when she presented with Jaccoud arthropathy, nephritis, and bullous eruptions on the face, scalp, and trunk. Laboratory analysis at the time showed a positive antinuclear antibody with a speckled pattern and positive anti-dsDNA antibodies. Since her diagnosis of SLE, the patient had been hospitalized twice for suspected immune-mediated complications of the CNS. On admission, the patient's initial examination revealed a left facial droop, left pronator drift, and decreased sensation to light touch in the distal left upper extremity. No lower extremity weakness or numbness was noted. Laboratory evaluation was remarkable for elevated erythrocyte sedimentation rate (40 mm/h), but normal levels of anti-dsDNA antibodies, C3, and C4. The brain MRI showed a new area of T2/fluid-attenuated inversion recovery (FLAIR) hyperintensity within the subcortical white matter of the right posterior central gyrus and associated heterogeneous faint enhancement (figure, A–C). The spine MRI was normal. CSF analyses were performed, demonstrating glucose 51 mg/dL, protein 21 mg/dL, erythrocytes 0/mm3, leukocytes 0/mm3, and immunoglobulin G (IgG) index 2110, and negative oligoclonal bands. Over the course of the following 4 days, the patient's condition rapidly deteriorated. She developed worsening left-sided weakness, now including the left lower extremity, a left homonymous hemianopia, and dysarthria. Multiple brain MRIs over this time course showed markedly worsening signal abnormality throughout the right cerebral hemisphere involving multiple lobes (figure, D–F).

  - **What would be your first differential diagnosis for this presentation and explain why (pros and cons)?**

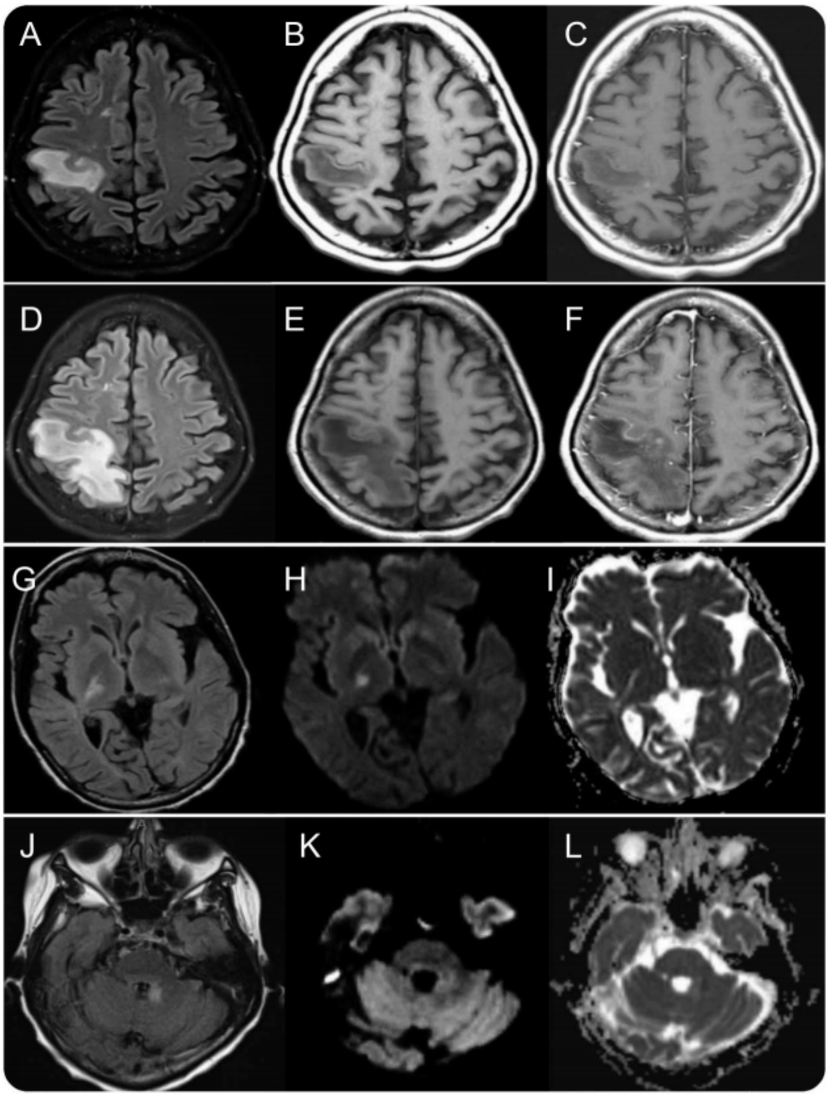

- **Ancillary exams results and follow-up:**

Negative studies for Gram stain, culture, venereal disease research laboratory, herpes simplex virus, Borrelia burgdorferi, and JC virus in CSF. Extensive bloodwork was performed. AntidsDNA was negative, anti-SS-A and anti-SS-B titers were elevated but at the patient's baseline, and the C3 and C4 levels remained roughly within normal ranges. The antiphospholipid panel and anti-rib-P antibodies were also within normal ranges. HIV test was negative. Although other tests, including brain biopsy, were pending at this point, given that the patient's condition was rapidly deteriorating and infectious etiologies were determined to be less likely, the patient was treated with the following immunomodulatory therapies: high-dose IV methylprednisolone, IV immunoglobulin (IVIg), and 1 dose of cyclophosphamide. The initial brain biopsy report demonstrated inflammation with prominent neutrophils and no evidence of malignancy or viral infection, but was inconclusive for any specific diagnosis. Several weeks later, the serum IgG anti-aquaporin-4 returned positive.

  - **What is your final diagnosis and explain why?**

Case 4

- **Initial presentation**

A 65-year-old man with well-controlled diabetes presented with 3 months of progressive leftside predominant weakness accompanied by painless paresthesias of his bilateral upper and lower extremities. The onset was with tingling and numbness in his toes, which later involved his fingers. Over a few weeks, he developed progressive weakness of his left lower extremity followed by the right lower extremity and later involvement of both upper extremities. By the time of presentation, the patient was unable to sit or walk without support and reported weight loss of 35 pounds over 3 months. The patient denied any toxic substance exposure, insect bites, recent illness or travel, family history of neuropathy, or any episodes suggestive of recurrent compressive neuropathies. His examination showed moderately severe asymmetric weakness (Medical Research Council scale: 3–4/5 on the left, 4–5/5 on the right) of the upper and lower extremities (distal more than proximal). There was asymmetric muscle atrophy; however, deep tendon reflexes were 2+ in the right biceps and triceps, 1+ in the left biceps, triceps, and bilateral ankles, and absent in the knees bilaterally. The sensory examination revealed left-sided predominant loss of pinprick in a patchy distribution in legs and loss of vibration sense in the toes. The remaining neurologic examination was unremarkable.

  - **What would be your first differential diagnosis for this presentation and explain why (pros and cons)?**

- **Ancillary exams results and follow-up:**

Blood tests revealed unremarkable complete blood count, comprehensive metabolic panel, mildly reduced vitamin B12 (163 pg/mL), normal methylmalonic acid (0.20 µmol/L), hemoglobin A1c 6.1%, creatine kinase 91 IU/L, erythrocyte sedimentation rate 18 mm/h, negative antinuclear antibodies, M protein, and nonreactive HIV I and II antibodies. CSF analysis revealed an elevated cell count of 22/µL (lymphocytic 89%) and elevated protein 127 mg/dL. MRI of the brain and entire spine with and without contrast was unremarkable except for mild degenerative disc disease in cervical and lumbar spine without any nerve root enhancement. Nerve conduction studies revealed conduction block in the forearm of the bilateral ulnar and left median nerves and mildly slowed conduction velocity of the left median and ulnar motor nerves (figure and tables). The left superficial peroneal sensory response was absent. The left sural, radial, and right ulnar sensory responses showed mildly reduced amplitudes and conduction velocities (table). F-wave latencies were prolonged in the left ulnar nerve (table). Needle EMG showed widespread active denervation in upper and lower extremities on the left side and abnormal spontaneous activity (fibrillation potentials and positive sharp waves) from cervical and lumbar paraspinal muscles (table). Nerve conduction studies showed conduction block in left ulnar and median nerves in the forearm, along with mildly slowed sensory velocities of bilateral ulnar and left sural and right median nerve. Sensory nerve action potential amplitudes were mildly

reduced in multiple nerves (bilateral median and radial, right ulnar, and left sural). The F-wave latency of left ulnar nerve was also prolonged (;130% of normal). Needle EMG showed neuropathic units in L4/5, L5/S1, and C7-T1-innervated muscles. The patient's recent weight loss and mildly elevated CSF cell count >10/µL prompted further workup for systemic disease. Contrast-enhanced CT of the chest, abdomen, and pelvis showed lymphadenopathy in the mediastinum, upper abdomen, and hilar regions.

- **What is your final diagnosis and explain why?**

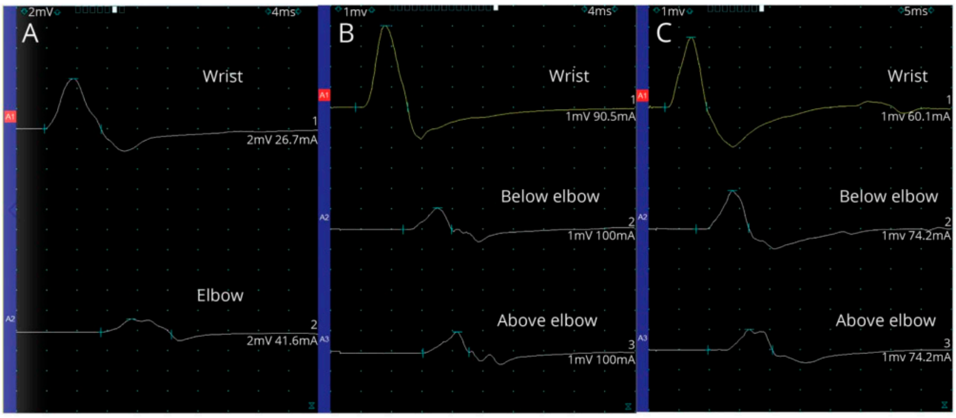

Nerve stimulated	Stimulation site	Recording site	Latency, ms	Amplitude (motor mV, sensory μV)	Conduction velocity, m/s	F wave, ms
**Motor NCS**						
**L median**	Wrist	APB	3.7	5		33.2
	Elbow	APB	11.2	1.3	36	
**R median**	Wrist	APB	3.3	10		22.9
	Elbow	APB	9.0	9.7	48	
**L ulnar**	Wrist	ADM	2.6	2.6		42.2
	Below elbow	ADM	7.9	1.3	51	
	Above elbow	ADM	9.8	1.3	51	
**L ulnar**	Wrist	FDI	3.3	4.0		
	Below elbow	FDI	9.5	1.1	39	
	Above elbow	FDI	12.1	1.1	38	
**R ulnar**	Wrist	ADM	2.6	3.5		26.9
	Below elbow	ADM	7.8	1.9	50	
	Above elbow	ADM	9.7	1.0	51	
**L peroneal**	Ankle	EDB	4.3	4.5		53.2
	Fibular head	EDB	10.1	3.6	57	
	Popliteal fossa	EDB	12.2	3.7	53	
**L tibial**	Ankle	AH	4.0	3.8		54.5
	Popliteal fossa	AH	12	3.0	53	
**Sensory NCS**						
**L median**	Wrist	Digit II	2.8	10.9	52	
**R median**	Wrist	Digit II	3.2	13.4	47	
**L ulnar**	Wrist	Digit V	2.7	16.3	45	
**R ulnar**	Wrist	Digit V	2.9	6.3	45	
**L radial**	Forearm	Anatominal snuff box	2.0	7.4	54	
**R radial**	Forearm	Anatominal snuff box	1.7	10.6	64	
**L sural**	Ankle	Lower leg	3.7	3.8	38	
**L superficial peroneal**	Ankle	Dorsum of foot	NR	NR	NR	

	Insertional	Spontaneous activity			Motor unit morphology			Recruitment		
**Muscle**	**IA**	**Fibrillation/ positive waves**	**Fasciculation**	**Other**	**Duration**	**Amplitude**	**Polyphasic**	**Number of motor unit action potentials**	**Firing rate**	**Activation**
**L deltoid**	Normal	None	None	None	Normal	Normal	Normal	Normal	Normal	Full
**L triceps brachii**	Normal	+/−	None	None	Normal	Normal	Normal	Mildly reduced	Normal	Full
**L flexor carpi radialis**	Normal	None	None	None	Normal	Normal	Normal	Normal	Normal	Full
**L flexor carpi ulnaris**	Increased	2+	None	None	Normal	Normal	25%–50%	Mildly reduced	Normal	Full
**L first dorsal interosseous**	Increased	+/−	None	None	Normal	Normal	Normal	Moderately reduced	Increased	Full
**L extensor indicis proprius**	Normal	None	None	None	Normal	Normal	Normal	Normal	Normal	Full
**L extensor pollicis longus**	Normal	None	None	None	Normal	Normal	25%–50%	Mildly reduced	Increased	Full
**L tibialis anterior**	Normal	None	None	None	Normal	Normal	Normal	Mildly reduced	Normal	Full
**L gastrocnemius**	Normal	None	None	None	Normal	Normal	Normal	Mildly reduced	Normal	Full
**L tibialis posterior**	Increased	1+	None	None	Normal	Normal	25%–50%	Moderately reduced	Increased	Full
**L vastus lateralis**	Increased	2+	None	None	Normal	Normal	25%–50%	Moderately reduced	Normal	Full
**L adductor longus**	Increased	1+	None	None	Normal	Normal	Normal	Mildly reduced	Normal	Full
**L gluteus medius**	Normal	None	None	None	Normal	Normal	Normal	Normal	Normal	Full
**L lumbar paraspinalis**	Increased	1+	None	None						
**L cervical paraspinalis**	Increased	1+	None	None						

Case 5

- **Initial presentation**

A 55-year-old man with an extensive psychiatric history that included PTSD, anxiety, depression, and schizophrenia treated with quetiapine and trazodone was admitted to the hospital from an assisted living facility (ALF) for subacute-on-chronic failure to thrive and concern for psychosis, with recent worsening ongoing for 6 weeks. He was recently discharged from an outside psychiatric facility, and at the ALF, he demonstrated odd behavior such as eating food off the floor and spitting on staff, in addition to persistent poor intake of fluids, food, and medications because of issues with regurgitation and dysphagia. He had been evaluated by gastroenterology for more than a decade for these issues, in addition to chronically elevated aspartate aminotransferase (AST) and alanine aminotransferase (ALT). An extensive workup including CT imaging, esophagogastroduodenoscopy, esophagram, motility study, and a gastric emptying study was largely unremarkable. In the setting of his psychiatric history and reported history of frequently spitting out food, there was concern for a volitional component to his regurgitation. Before hospitalization, he had been seen by outpatient neurologists for hyperkinetic movements and was given the diagnosis of Tourette syndrome at age 40 years. A diagnosis of akathisia was entertained because he had been on quetiapine for psychiatric symptoms for many years. During this admission, the neurologic examination was notable for a thin man with frequent hyperkinetic movements affecting the trunk and bilateral upper extremities with a random and flowing quality, most prominent when sitting up. He denied experiencing an urge to move, and he could not suppress the movements. There was a mild milkmaid grip, and he was unable to maintain the tongue fully protruded for 5 seconds. Otherwise, his examination demonstrated normal extraocular movements, intact sensation to multiple modalities, and no overt appendicular ataxia. Deep tendon reflexes were difficult to assess because he would not cooperate with the examination. He was able to sit unassisted and walk unassisted, although he exhibited a staggering gait and his tandem gait was unsteady. He denied having a family history of similar symptoms. Laboratory workup was notable for a creatinine of 1.6 mg/dL (baseline 1.2 mg/dL) and elevated AST (304 U/L) and ALT (154 U/L).

  - **What would be your first differential diagnosis for this presentation and explain why (pros and cons)?**

- **Ancillary exams results and follow-up:**

Serum copper and ceruloplasmin tests were recommended. These tests were both in the normal range. Because of his chronic transaminitis (elevated AST and ALT), an extensive gastroenterologic and rheumatologic workup was performed but was unrevealing. Because the transaminitis could indicate muscle breakdown and liver pathology, a creatine kinase (CK) level was checked and was found to be elevated at 4,753 IU/L. From a chart review of outside records, his baseline CK ranged from 600 to 800 IU/L. His CK returned to his previous elevated baseline with the administration

of intravenous fluids. MRI brain with and without contrast was recommended, given his long-standing abnormal movements to assess for a structural etiology or evidence of a neurodegenerative process. This revealed atrophy of the bilateral caudate (Figure).

- **What is your final diagnosis and explain why?**

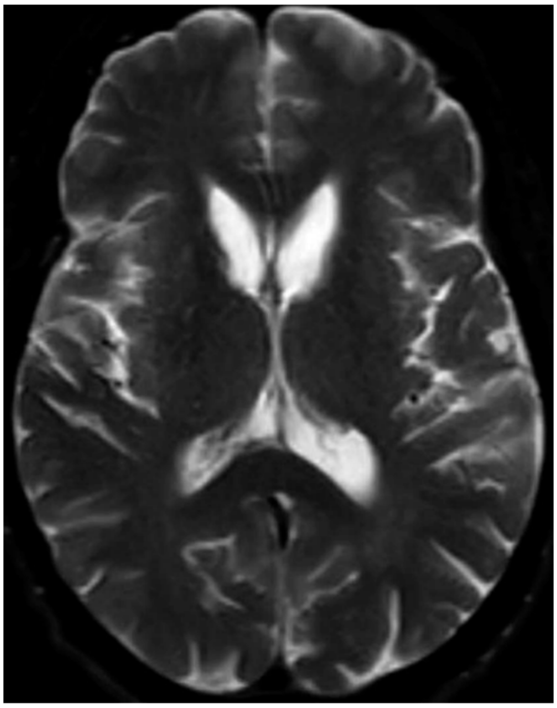

Supplementary S2. Scoring sheets

Case 1

Q1 - Differential diagnosis:

- Inflammatory vasculopathy:
  - Medium-vessel vasculitis: Polyarteritis (/1)
  - Large-vessel vasculitis: Giant cell arteritis (/1)
  - Primary angiitis of the CNS (/1)
- Non-inflammatory vasculopathy:
  - Intracranial atherosclerotic disease (/1)
- Neoplastic/paraneoplastic vasculopathy
  - Lymphoproliferative disorders (/1)
  - Paraneoplastic syndromes (/1)
- Penalties point for aberrancy or potential harmful hypothesis (/2) (1 point per diagnosis, if >2 clinical cases are failed).
- Bonuses for plausible and correctly justified alternate diagnosis (/2)

NB: points are awarded only with a valid justification.

Q2 – Final diagnosis:

- Intravascular lymphoma (/3)
- Diffuse B-cell lymphoma (/1)
- Penalties point for aberrancy or potential harmful hypothesis (/2) (1 point per diagnosis, if >2 clinical cases are failed)

Case 2

Q1 - Differential diagnosis:

- Vasculitis
    - PACNS (/1)
    - Secondary causes (e.g., Lupus, Sjögren, Wegener, Bechet, Susac) (/1)
- ADEM or post-infectious encephalomyelitis (/1).

- Penalties points for aberrancy or potential harmful hypothesis (/2) (1 point per diagnosis, if >2 clinical cases are failed).
- Bonuses for plausible and correctly justified alternate diagnosis (/2)

NB: points are awarded only with a valid justification.

Q2 – Final diagnosis:

- Susac syndrome (/3)
- Vasculitis of any nature (/1)
- Penalties point for aberrancy or potential harmful hypothesis (/2) (1 point per diagnosis, if >2 clinical cases are failed)

Case 3

Q1 - Differential diagnosis:

- Multiple sclerosis (/1).
- Multiple sclerosis variants (e.g., Shilder, Marburg, Balo) (/1).
- NMOSD (/1).
- ADEM (/1).
- Progressive multifocal leukoencephalopathy (/1).
- Drug-induced leukoencephalopathy (e.g., MTX-induced) (/1).
- Malignancies (e.g., lymphoma, high-grade glioma) (/1).

- Penalties points for aberrancy or potential harmful hypothesis (/2) (1 point per diagnosis, if >2 clinical cases are failed).
- Bonuses for plausible and correctly justified alternate diagnosis (/2)

NB: points are awarded only with a valid justification.

Q2 – Final diagnosis:

- NMOSD (/3)
- CNS demyelinating disease (/1)
- Penalties points for aberrancy or potential harmful hypothesis (/2) (1 point per diagnosis, if >2 clinical case is failed)

Case 4

Q1 - Differential diagnosis:

- Inflammatory/autoimmune:
  - Vasculitis (/1).
  - CIDP (/1).
- Hereditary:
  - Hereditary neuropathy with liability to pressure palsy (/1).
- Infectious (e.g., HIV, Lyme, hepatitis) (/1).
- Malignancy (e.g., lymphoma). (/1).
- Paraneoplastic syndrome. (/1).
- Systemic diseases (e.g., sarcoidosis, amyloidosis) (/1).

- Penalties points for aberrancy or potential harmful hypothesis (/2) (1 point per diagnosis, if >2 clinical cases are failed).
- Bonuses for plausible and correctly justified alternate diagnosis (/2)

NB: points are awarded only with a valid justification.

Q2 – Final diagnosis:

- Neurosarcoidosis (/3)
- Paraneoplastic neuropathy (/3)
- Penalties points for aberrancy or potential harmful hypothesis (/2) (1 point per diagnosis, if >2 clinical cases are failed)

Case 5

Q1 - Differential diagnosis:

- **Basal ganglia structural disorders (e.g. vascular or demyelinating) (/1).**
- **Toxic-metabolic disorders (e.g., hyperglycemia, electrolytes derangements) (/1).**
- **Drug-induced (e.g., dopaminergic therapy, tardive dyskinesia) (/1).**
- **Systemic auto-immune processes (e.g., lupus, APLS) (/1).**
- **Hereditary disorders (e.g., Huntington, neuroacanthocytosis, Wilson) (/1).**
- **Paraneoplastic syndromes (e.g., anti-CRMP5 or anti-NMDA) (/1).**

- Penalties points for aberrancy or potential harmful hypothesis (/2) (1 point per diagnosis, if >2 clinical cases are failed).
- Bonuses for plausible and correctly justified alternate diagnosis (/2)

NB: points are awarded only with a valid justification.

Q2 – Final diagnosis:

- Neuroacanthocytosis (/3)
- Huntington disease (/1)
- Penalties points for aberrancy or potential harmful hypothesis (/2) (1 point per diagnosis, if >2 clinical case is failed)

Supplementary S3. AI Sourced Replies

Case 1 - Q1

1. Cerebral Autosomal Dominant Arteriopathy with Subcortical Infarcts and Leukoencephalopathy (CADASIL): Pros - Could explain the multifocal constriction of the middle cerebral arteries and the multiple foci of restricted diffusion. Cons - Typically presents earlier in life and is associated with migraine and psychiatric disturbances, which are not reported in this case.

2. Cerebral Amyloid Angiopathy (CAA): Pros - More common in elderly patients, which fits with the patient's age. Cons - MRI does not show the typical lobar microbleeds associated with CAA.

3. Vasculitis: Pros - Could cause the multifocal constriction of the middle cerebral arteries seen on CT angiogram. Cons - Patient does not have any systemic symptoms such as fever, malaise, or weight loss that are typically associated with vasculitis.
    1. Primary Central Nervous System Vasculitis (PCNSV): Pros - Could cause multiple strokes. Cons - Usually presents with headache, cognitive dysfunction, or focal neurologic deficits.
    2. Giant Cell Arteritis (Temporal Arteritis): Pros - Can cause stroke. Cons - Usually associated with headache, jaw claudication, and visual disturbances.
    3. Polyarteritis Nodosa: Pros - Can affect any organ, including the brain. Cons - Usually associated with systemic symptoms such as weight loss, fever, and malaise.
    4. Wegener's Granulomatosis: Pros - Can affect small to medium-sized vessels in many organs, including the brain. Cons - Usually associated with respiratory or renal symptoms.
    5. Behçet's Disease: Pros - Can cause neurologic symptoms due to inflammation of the brain's blood vessels. Cons - Usually associated with oral and genital ulcers and eye inflammation.
    6. Vasculitis associated with Connective Tissue Diseases: Pros - Conditions such as Systemic Lupus Erythematosus or Rheumatoid Arthritis can be associated with vasculitis that affects the brain. Cons - Patient does not have any reported symptoms of these conditions.

4. Stroke due to Cardioembolic or Large Vessel Atherosclerotic Disease: Pros - Patient's age and the presence of multiple infarcts of varying ages. Cons - Multifocal constriction of the middle cerebral arteries is not typical for these conditions.

5. Reversible Cerebral Vasoconstriction Syndrome (RCVS): Pros - Characterized by a reversible narrowing of the cerebral arteries, which could explain the

multifocal constriction seen on CT angiogram. Cons - Typically presents with a thunderclap headache, which is not reported in this case.

6. Moyamoya Disease: Pros - Could potentially cause multiple infarcts in different vascular territories. Cons - Typically affects children and the "puff of smoke" appearance on angiography is not reported in this case.
7. Cryptogenic Stroke: Pros - Could be a possibility if no clear cause of stroke can be identified. Cons - The multifocal constriction of the middle cerebral arteries seen in this patient suggests a specific underlying pathology.
8. Intracranial Atherosclerotic Disease (ICAD): Pros - Could potentially cause multiple infarcts in different vascular territories. Cons - Patient's imaging findings suggest a vasculitic or vasospastic process rather than atherosclerosis.
9. Hypercoagulable States: Pros - Could lead to multiple strokes. Cons - Less likely in the absence of a personal or family history of thrombosis.
10. Infectious or Paraneoplastic Causes: Pros - Certain infections or malignancies can cause vasculitis leading to stroke. Cons - Less likely in the absence of other systemic symptoms or risk factors.

Bibliography:

Daroff, R. B., Jankovic, J., Mazziotta, J. C., Pomeroy, S. L., Bradley, W. G. (2016). Bradley's neurology in clinical practice. Elsevier. Page No: 1181. DOI: 0323339166.

Daroff, R. B., Jankovic, J., Mazziotta, J. C., Pomeroy, S. L., Bradley, W. G. (2016). Bradley's neurology in clinical practice. Elsevier. Page No: 1051. DOI: 0323339166.

Rowland, L. P., Pedley, T. A., Merritt, H. H. (2016). Merritt's neurology. Wolters Kluwer. Page No: 854. DOI: 145119336X.

⇒ 3/3 relevant – no hallucinations

Case 1 - Q2

The final diagnosis for this patient is intravascular large B-cell lymphoma (IVLBCL). This is a rare type of non-Hodgkin lymphoma characterized by the selective growth of lymphoma cells within the lumen of small blood vessels, particularly capillaries, leading to vascular occlusion and tissue damage.

The diagnosis is supported by the patient's clinical presentation, imaging findings, and laboratory results. The patient's recurrent strokes with new ischemic lesions despite high-dose steroid and cyclophosphamide treatment, along with an angiogram showing a "string of beads" pattern in small and medium-sized vessels, are indicative of vasculopathy. Elevated inflammatory markers and the presence of identical oligoclonal immunoglobulin G bands in both serum and cerebrospinal fluid (CSF) suggest an inflammatory process and increased blood-brain barrier permeability.

The key finding pointing to IVLBCL is the bone marrow biopsy, which shows a low level of patchy interstitial infiltration by abnormal large B-cells, consistent with large B-cell lymphoma, with dual expression of C-MYC and BCL2. Additionally, CD34 staining reveals focal areas suggestive of possible intravascular involvement, which strongly supports the diagnosis of IVLBCL.

Diagnosing IVLBCL is often challenging due to its non-specific clinical presentation and the absence of detectable circulating lymphoma cells in peripheral blood. It is typically not associated with an obvious extravascular tumor mass or lymphadenopathy, which aligns with the patient's CT scan findings showing no solid organ malignancy or lymph node enlargement.

In conclusion, the patient's clinical presentation, imaging findings, and laboratory results—especially the bone marrow biopsy findings—support a diagnosis of intravascular large B-cell lymphoma. This diagnosis explains the patient's recurrent strokes, vasculopathy, elevated inflammatory markers, and lack of response to high-dose steroid and cyclophosphamide treatment.

References:

Ferreri, A. J., Campo, E., Seymour, J. F., Willemze, R., Ilariucci, F., Ambrosetti, A., ... & Zucca, E. (2004). Intravascular lymphoma: clinical presentation, natural history, management and prognostic factors in a series of 38 cases, with special emphasis on the 'cutaneous variant'. British journal of haematology, 127(2), 173-183. Page No: 512, 515. DOI: 10.1111/j.1365-2141.2004.05177.x.

⇒ 1/1 relevant – no hallucinations

Case 2 - Q1

Multiple Sclerosis (MS): The patient's MRI findings of multiple non-enhancing T2 hyperintense lesions in the supratentorial white matter, periventricular white matter, and corpus callosum, along with the presence of oligoclonal bands in the CSF, are

suggestive of MS. However, the absence of clinical signs of optic neuritis, spinal cord symptoms, and the presence of gaze-evoked nystagmus make MS less likely.

Sneddon Syndrome: This condition, characterized by livedo reticularis and cerebrovascular disease, could explain the patient's cognitive decline and MRI findings. However, the absence of livedo reticularis and the presence of gaze-evoked nystagmus do not support this diagnosis.

Susac Syndrome: This is a rare disorder characterized by the triad of encephalopathy, branch retinal artery occlusion, and hearing loss, which matches some of the patient's symptoms and findings. The MRI findings of T2 hyperintense lesions in the corpus callosum are also seen in Susac syndrome. However, the patient's age (it usually affects people aged 20-40) and the absence of typical "snowball" MRI lesions make this diagnosis less likely.

Cerebral Amyloid Angiopathy (CAA): This condition can cause cognitive decline and cerebral microbleeds, which could explain the patient's symptoms and MRI findings. However, the patient's relatively young age and the absence of lobar, cortical, or cortico-subcortical cerebral microbleeds make CAA less likely.

Vasculitis: This could cause cognitive decline and multiple cerebral lesions. However, the absence of systemic symptoms such as fever, malaise, weight loss, and anemia, and the normal cell count in the CSF make this diagnosis less likely.
Primary CNS Vasculitis: This condition could explain the patient's cognitive decline and MRI findings. However, the absence of systemic symptoms and the normal cell count in the CSF make this diagnosis less likely. Primary CNS vasculitis typically presents with headaches, cognitive impairment, and focal neurologic deficits, and MRI often shows multiple infarcts or hemorrhages.
Systemic Vasculitis (e.g., Granulomatosis with Polyangiitis, Lupus): These conditions could also cause neurologic symptoms and MRI changes due to inflammation of the blood vessels in the brain. However, these conditions usually present with systemic symptoms and signs such as fever, weight loss, skin rash, joint pain, and kidney disease, which are not reported in this patient.

Infectious or Post-Infectious Encephalitis: Given the patient's recent upper respiratory symptoms, an infectious or post-infectious cause could be considered.
Viral Encephalitis (e.g., Herpes Simplex Virus, Varicella-Zoster Virus, Enteroviruses): These viruses can cause encephalitis with symptoms of cognitive decline and MRI changes. However, these conditions usually present with fever and CSF pleocytosis, which are not reported in this patient.
Post-Infectious or Autoimmune Encephalitis (e.g., Anti-NMDA Receptor Encephalitis, Anti-LGI1 Encephalitis): These conditions can cause subacute cognitive decline and MRI changes due to an autoimmune response triggered by a previous infection. However, these conditions often present with other neurologic symptoms such as seizures, movement disorders, and psychiatric symptoms, which are not reported in this patient.

Toxic or Metabolic Encephalopathy: The patient's positive toxicology screen for cannabinoids and amphetamines raises the possibility of a toxic or metabolic cause. However, the patient's cognitive decline persisted despite removal from the potential toxic environment, making this less likely.

Leukodystrophies: These are rare genetic disorders that affect the white matter of the brain. The MRI findings could be suggestive of a leukodystrophy. However, these conditions usually present in childhood or adolescence, making this diagnosis less likely in a 44-year-old patient.

Neoplastic or Paraneoplastic Syndromes: These could cause cognitive decline and multiple cerebral lesions. However, the absence of systemic symptoms such as weight loss, night sweats, and the absence of enhancement of the lesions on MRI make this diagnosis less likely.

Bibliography:

Daroff, R. B., Jankovic, J., Mazziotta, J. C., Pomeroy, S. L., Bradley, W. G. (2016). Bradley's neurology in clinical practice. Elsevier. DOI: 0323339166. Pages: 149, 564, 569, 1061, 1223.

Ropper. (2014). Adams and Victor's principles of neurology, 10e. McGraw-Hill. DOI: 9780071794794. Pages: 1543, 2032, 889.

⇒ 8/8 relevant – no hallucinations

Case 2 - Q2

The final diagnosis for this patient is Susac Syndrome.

Susac Syndrome is a rare disorder characterized by a clinical triad of encephalopathy, branch retinal artery occlusion (BRAO), and sensorineural hearing loss. In this patient, the presence of cognitive decline (encephalopathy), evidence of BRAO on ophthalmoscopic examination, and complaints of hearing loss align with this triad.

The MRI findings of T2 hyperintense lesions in the corpus callosum are also consistent with Susac Syndrome. Although the patient is slightly older than the typical age range for Susac Syndrome (which usually affects people aged 20-40), it can still occur in this age group.

The absence of enhancement of the lesions on MRI and the presence of oligoclonal bands in the CSF further support this diagnosis. The lack of findings on magnetic resonance angiography and cerebral catheter angiography help to exclude vasculitis.

The normal serologic studies, including antinuclear antibody assay, antineutrophil cytoplasmic antibody, and others, help to exclude autoimmune and infectious etiologies. The negative workup for other treatable causes of subacute cognitive decline, such as vitamin deficiency, anti-NMDA encephalitis, Hashimoto encephalitis, Wilson disease, infectious encephalitis, neurosarcoidosis, and thromboembolic disease, further supports the diagnosis of Susac Syndrome.

In conclusion, while Susac Syndrome is a rare condition, it is important to consider in cases of encephalopathy, BRAO, and hearing loss, especially when more common diagnoses have been excluded.

Bibliography:
Daroff, R. B., Jankovic, J., Mazziotta, J. C., Pomeroy, S. L., Bradley, W. G. (2016). Bradley's neurology in clinical practice. Elsevier. DOI: 0323339166. Pages: 237, 1294.
Rowland, L. P., Pedley, T. A., Merritt, H. H. (2016). Merritt's neurology. Wolters Kluwer. DOI: 145119336X. Page: 690.

⇒ 3/3 relevant – no hallucinations

Case 3 - Q1

1. Neuropsychiatric Systemic Lupus Erythematosus (NPSLE):

Pros: The patient's history of SLE and the presence of neurological symptoms could suggest NPSLE. The MRI findings of hyperintense lesions in the subcortical white matter are consistent with this diagnosis.
Cons: However, the patient's normal levels of anti-dsDNA antibodies, C3, and C4 are not typical for active NPSLE. Furthermore, the rapid progression of symptoms and the extensive involvement of the right cerebral hemisphere are not typical for NPSLE.

2. Multiple Sclerosis (MS):

Pros: The patient's symptoms of left-sided weakness and sensory loss, as well as the MRI findings of T2/FLAIR hyperintensity in the subcortical white matter, could suggest MS.
Cons: However, the patient's age and the rapid progression of symptoms are not typical for MS. Additionally, the absence of oligoclonal bands in the CSF is not consistent with MS.

3. Neuromyelitis Optica Spectrum Disorder (NMOSD):

Pros: The rapid progression of symptoms and the extensive involvement of the right cerebral hemisphere on MRI could suggest NMOSD.
Cons: However, the patient's normal spine MRI and the absence of optic neuritis are not typical for NMOSD. Furthermore, the patient's normal IgG index and the absence of oligoclonal bands in the CSF are not consistent with NMOSD.

4. CNS Vasculitis: Primary Angiitis of the Central Nervous System (PACNS) or Systemic Lupus Erythematosus (SLE)-related Vasculitis

Pros: The patient's history of SLE and the presence of neurological symptoms could suggest CNS vasculitis. The MRI findings of hyperintense lesions in the subcortical white matter are consistent with this diagnosis.
Cons: However, the patient's normal levels of anti-dsDNA antibodies, C3, and C4 are not typical for active CNS vasculitis. Furthermore, the rapid progression of symptoms and the extensive involvement of the right cerebral hemisphere are not typical for CNS vasculitis.

5. Progressive Multifocal Leukoencephalopathy (PML):

Pros: The patient's history of immunosuppressive therapy for SLE and the MRI findings of hyperintense lesions in the subcortical white matter could suggest PML.
Cons: However, the patient's normal CSF analysis and the absence of JC virus DNA in the CSF are not consistent with PML.

6. Autoimmune Encephalitis:
    1. Anti-NMDA Receptor Encephalitis: The rapid progression of symptoms and the extensive involvement of the right cerebral hemisphere on MRI could suggest Anti-NMDA Receptor Encephalitis. However, this condition often presents with psychiatric symptoms, memory problems, and seizures, which the patient does not have.
    2. Limbic Encephalitis: The patient's symptoms and MRI findings could suggest Limbic Encephalitis. However, this condition often presents with memory problems, seizures, and psychiatric symptoms, which the patient does not have.
    3. Anti-LGI1 Encephalitis: The patient's symptoms and MRI findings could suggest Anti-LGI1 Encephalitis. However, this condition often presents with memory problems, seizures, and movement disorders, which the patient does not have.
7. Neoplasms:
    1. Primary Central Nervous System Lymphoma (PCNSL): This is a rare form of non-Hodgkin lymphoma that is confined to the brain, spinal cord, or eye. The diagnosis is typically confirmed by brain biopsy.
    2. Metastatic Brain Tumors: Given the patient's age, metastatic brain tumors could be a potential cause of her symptoms. The diagnosis would typically be confirmed by brain biopsy or identification of a primary tumor elsewhere in the body.

Bibliography:
Daroff, R. B., Jankovic, J., Mazziotta, J. C., Pomeroy, S. L., Bradley, W. G. (2016). Bradley’s neurology in clinical practice. Elsevier. Pages 1192, 334, 1075, 570, 1361. DOI: 0323339166.
Ropper. (2014). Adams and Victor’s principles of neurology, 10e. McGraw-Hill. Page 1224. DOI: 9780071794794.

⇒ 6/6 relevant – no hallucinations

Case 3 - Q2

The final diagnosis for this patient is neuromyelitis optica spectrum disorder (NMOSD). This conclusion is based on the following reasons:

Clinical Presentation:

The patient's symptoms, including left-sided weakness, left homonymous hemianopsia, and dysarthria, are consistent with NMOSD. The rapid progression of symptoms and the involvement of multiple brain lobes are also characteristic of the disorder.

MRI Findings:

MRI results, including the large right hemispheric lesion in the precentral gyrus and the rapid progression of the lesion over a short period, align with NMOSD. Previous MRI findings from five and four years ago also suggest a history of NMOSD-related lesions.

Laboratory Findings:

A positive serum test for anti-aquaporin-4 IgG is a key diagnostic marker for NMOSD. The absence of other abnormal laboratory findings, including negative infectious disease tests and normal levels of anti-dsDNA antibodies, C3, and C4, further supports the diagnosis.

Response to Treatment:

The patient's response to immunomodulatory therapies, including high-dose IV methylprednisolone, IV immunoglobulin (IVIg), and cyclophosphamide, is consistent with the standard treatment approach for NMOSD.

Brain Biopsy:

Although the brain biopsy was inconclusive for a specific diagnosis, the absence of malignancy or viral infection helps rule out other potential diagnoses.

Bibliography:
Daroff, R. B., Jankovic, J., Mazziotta, J. C., Pomeroy, S. L., Bradley, W. G. (2016). Bradley's neurology in clinical practice. Elsevier. Pages 1256-1257. DOI: 0323339166.

⇒ 2/2 relevant – no hallucinations

Case 4 - Q1

Diabetic Polyneuropathy: Given the patient's history of well-controlled diabetes, the progressive, predominantly distal weakness, and sensory loss, diabetic

polyneuropathy is a strong possibility. This condition is characterized by distal sensory loss and weakness in the limbs, as well as areflexia. The patient's weight loss and painless paresthesias are also consistent with this diagnosis. However, the asymmetry of symptoms and the patchy distribution of sensory loss are less typical for a symmetric polyneuropathy and may suggest an alternative or additional diagnosis.

Diabetic Lumbosacral Radiculoplexopathy: This condition, also known as diabetic amyotrophy, typically presents with unilateral severe pain in the lower back, hip, and anterior thigh, followed by weakness. Weight loss occurs in more than half of patients. The patient's presentation is somewhat atypical, given the bilateral and upper extremity involvement, and the absence of pain.

Multiple Mononeuropathies: This condition involves simultaneous or sequential damage to two or more noncontiguous peripheral nerves, which can simulate a length-dependent peripheral polyneuropathy. The patient's asymmetric presentation could be consistent with this diagnosis. However, the progression of symptoms from distal to proximal is less typical for this condition.

Vasculitic Neuropathy: This condition can cause asymmetric deficits due to multiple infarcts, which may later coalesce, causing symmetric deficits. The patient's weight loss could be consistent with a systemic vasculitic process. However, the absence of other systemic symptoms makes this less likely.

Chronic Inflammatory Demyelinating Polyneuropathy (CIDP): This autoimmune condition can cause progressive weakness and sensory loss. However, it typically presents with more symmetric symptoms and often includes more prominent proximal weakness.

Paraneoplastic Neuropathy: Paraneoplastic syndromes can cause a variety of neurologic symptoms, including peripheral neuropathy. These syndromes occur when cancer-fighting antibodies or white blood cells (known as T cells) mistakenly attack normal cells in the nervous system. The patient's age and unexplained weight loss could potentially point towards a malignancy. However, the absence of other systemic symptoms makes this less likely.

Systemic Lupus Erythematosus (SLE): SLE can cause a variety of neurologic manifestations, including peripheral neuropathy. However, the patient's presentation is not typical for SLE, which often includes other systemic symptoms such as rash, arthritis, and renal involvement.

Sarcoidosis: Neurosarcoidosis can cause peripheral neuropathy, but it is relatively rare and usually associated with systemic symptoms of sarcoidosis, which are not reported in this case.

Lyme Disease: Lyme disease can cause a peripheral neuropathy, often manifesting as a painful radiculoneuritis. However, the patient denied any recent travel or insect bites, making this less likely.

HIV: HIV can cause a distal symmetric polyneuropathy, among other neurologic manifestations. Given the patient's age and lack of other risk factors, this seems less likely, but could be considered if the patient has risk factors for HIV infection.

Toxic or Metabolic Neuropathy: Although the patient denied any toxic exposure, it would be important to consider potential unrecognized exposures or a metabolic cause, such as renal failure. However, the patient's well-controlled diabetes makes a separate metabolic cause less likely.

Bibliography:
Bradley, W. G., Daroff, R. B., Jankovic, J., Mazziotta, J. C., Pomeroy, S. L. (2016). Bradley's neurology in clinical practice. Elsevier. DOI: 0323339166. Pages: 1067, 2243, 2323, 2324, 2325, 2330, 2341, 338.
Rowland, L. P., Pedley, T. A., Merritt, H. H. (2016). Merritt's neurology. Wolters Kluwer. DOI: 145119336X. Page: 1472.

⇒ 9/9 relevant – no hallucinations

Case 4 - Q2

The patient's clinical presentation, laboratory findings, and imaging results suggest a diagnosis of Paraneoplastic Neuropathy.

Here's why:

The patient's nerve conduction studies and electromyography results show evidence of a peripheral neuropathy with conduction block in the forearm of the bilateral ulnar and left median nerves, mildly slowed conduction velocity of the left median and ulnar motor nerves, and widespread active denervation in upper and lower extremities on the left side.

The patient's CSF analysis revealed an elevated cell count and protein level, which can be seen in paraneoplastic syndromes.

The patient's recent weight loss and mildly elevated CSF cell count prompted further workup for systemic disease. The contrast-enhanced CT of the chest, abdomen, and pelvis showed lymphadenopathy in the mediastinum, upper abdomen, and hilar regions, which could suggest an underlying malignancy causing a paraneoplastic syndrome.

The patient's mildly reduced vitamin B12 level could potentially contribute to the neuropathy, but the normal methylmalonic acid level makes a B12 deficiency less likely as the primary cause.

In conclusion, while the patient's presentation is complex and could potentially be explained by several different conditions, the combination of peripheral neuropathy, elevated CSF cell count and protein level, and systemic signs of malignancy suggest a

paraneoplastic neuropathy. Further workup, including potentially a biopsy of the lymph nodes, would be necessary to confirm the diagnosis and identify the underlying malignancy.

Bibliography:
Bradley, W. G., Daroff, R. B., Jankovic, J., Mazziotta, J. C., Pomeroy, S. L. (2016). Bradley's neurology in clinical practice. Elsevier. DOI: 0323339166. Pages: 2337, 2312, 2339.
Rowland, L. P., Pedley, T. A., Merritt, H. H. (2016). Merritt's neurology. Wolters Kluwer. DOI: 145119336X. Page: 1180.

⇒ 4/4 relevant – no hallucinations

Case 5 - Q1

Drug-induced movement disorders (DIMDs):
Given the patient's long-term use of quetiapine, a type of antipsychotic medication, it is possible that he has developed DIMDs. These can include tardive dyskinesia, akathisia, and parkinsonism, all of which can present with hyperkinetic movements. The pros of this diagnosis are that it fits well with the patient's medication history and movement symptoms. The cons are that it doesn't explain the patient's gastrointestinal symptoms or elevated liver enzymes.

Neuroacanthocytosis syndromes:
These are a group of rare, genetic neurodegenerative disorders that can present with a variety of symptoms, including movement disorders, psychiatric symptoms, and dysphagia. The pros of this diagnosis are that it could potentially explain the patient's movement and gastrointestinal symptoms. The cons are that it's a rare condition and doesn't account for the patient's elevated liver enzymes.

Hepatocerebral degeneration:
This is a condition that can occur in patients with chronic liver disease, leading to neurological symptoms such as movement disorders and cognitive changes. The pros of this diagnosis are that it could explain the patient's movement symptoms and elevated liver enzymes. The cons are that it doesn't fully account for the patient's psychiatric or gastrointestinal symptoms.

Neurological complications of a systemic disease:
Given the patient's elevated liver enzymes and creatinine, it's possible that a systemic disease, such as liver disease or kidney disease, could be causing his neurological symptoms. The pros of this diagnosis are that it could potentially explain all of the patient's symptoms. The cons are that it would require further investigation to identify the underlying systemic disease.

Functional neurological disorder (FND):

FND is a condition in which patients experience neurological symptoms that are not explained by a traditional neurological disease. Given the patient's extensive psychiatric history and the presence of atypical symptoms (such as the inability to suppress movements), FND could be a possibility. The pros of this diagnosis are that it could potentially explain all of the patient's symptoms. The cons are that FND is a diagnosis of exclusion, meaning it should only be considered after other potential causes have been ruled out.

In conclusion, this patient's presentation is complex and could potentially be explained by a variety of neurological conditions. Further diagnostic testing, including genetic testing, liver and kidney function tests, and potentially a brain MRI, may be helpful in narrowing down the differential diagnosis.

Bibliography:
Daroff, R. B., Jankovic, J., Mazziotta, J. C., Pomeroy, S. L., Bradley, W. G. (2016). Bradley's neurology in clinical practice. Elsevier. Page No: 304, 1828, 1890. DOI: 0323339166
Rowland, L. P., Pedley, T. A., Merritt, H. H. (2016). Merritt's neurology. Wolters Kluwer. Page No: 1348. DOI: 145119336X
Jung, H. H., Danek, A., Walker, R. H. (2011). Neuroacanthocytosis Syndromes. Orphanet Journal of Rare Diseases, 6, 68. DOI: 10.1186/1750-1172-6-68

⇒ 5/5 relevant – no hallucinations

Case 5 - Q2

The patient's clinical presentation and ancillary exam results suggest two primary diagnostic possibilities: Huntington's disease and Chorea-acanthocytosis (ChAc).

Huntington's disease: This is a genetic disorder characterized by progressive brain cell death, leading to movement disorders, psychiatric symptoms, and cognitive decline. The patient's chronic movement disorder, psychiatric history, elevated creatine kinase (CK) level, and bilateral caudate atrophy on MRI are all consistent with Huntington's disease. Although the lack of a family history of similar symptoms makes Huntington's disease less likely, it can occur sporadically without a known family history. Genetic testing for the Huntington gene (HTT) would provide a definitive diagnosis.

Chorea-acanthocytosis (ChAc): This is a rare neurodegenerative disorder characterized by movement disorders, cognitive and behavioral changes, and acanthocytosis. The patient's movement disorder, psychiatric symptoms, elevated CK level, and bilateral caudate atrophy on MRI could also be consistent with ChAc. However, ChAc is a very rare condition and less likely than Huntington's disease. Genetic testing for mutations in the VPS13A gene would provide a definitive diagnosis for ChAc.

Given the patient's clinical presentation and the results of the ancillary exams, Huntington's disease is the more probable diagnosis. This is primarily due to the characteristic clinical features and the fact that Huntington's disease is a more common cause of chorea and psychiatric symptoms than ChAc. However, a definitive diagnosis would require genetic testing for both conditions.

Bibliography:
Daroff, R. B., Jankovic, J., Mazziotta, J. C., Pomeroy, S. L., Bradley, W. G. (2016). Bradley's neurology in clinical practice. Elsevier. Page No: 304, 1890. DOI: 0323339166

Rowland, L. P., Pedley, T. A., Merritt, H. H. (2016). Merritt's neurology. Wolters Kluwer. Page No: 1445. DOI: 145119336X

Jung, H. H., Danek, A., Walker, R. H. (2011). Neuroacanthocytosis Syndromes. Orphanet Journal of Rare Diseases, 6, 68. DOI: 10.1186/1750-1172-6-68

⇒ 4/4 relevant – no hallucinations

Supplementary Figure S1. Normal Q-Q plot

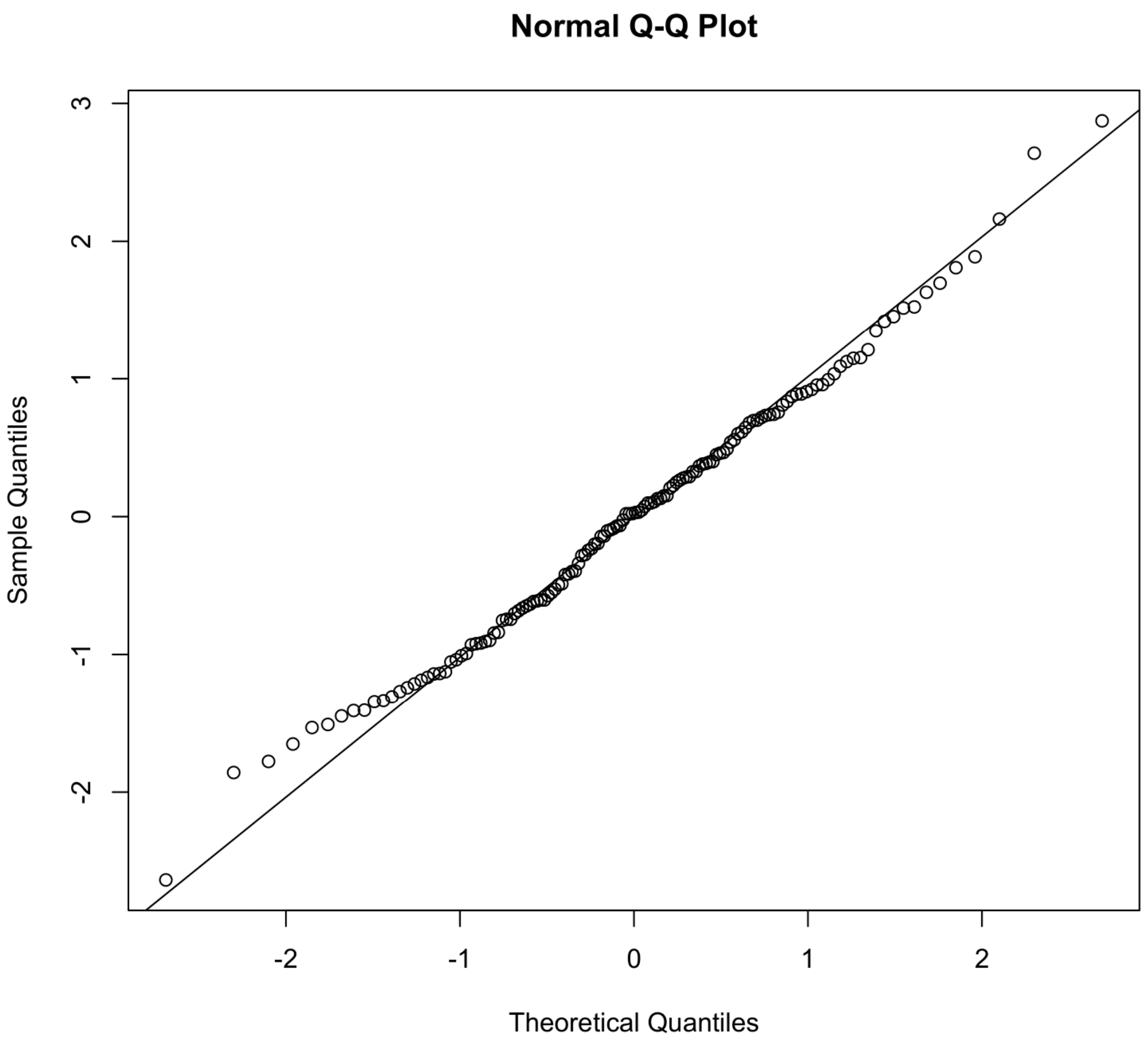

Supplementary Figure S2. Plot of residuals

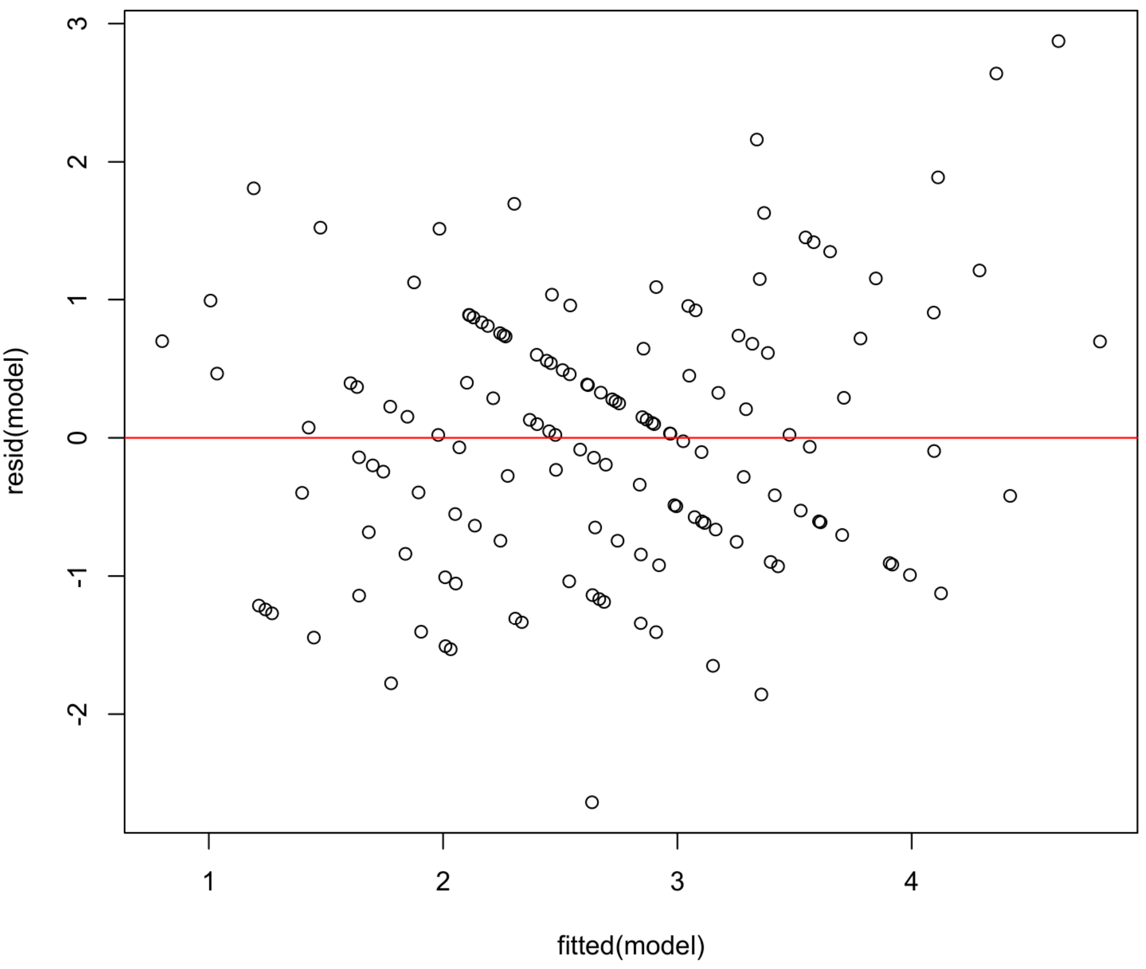

Supplementary S4. AI's null scores

Case 2

Question 2.1 – AI's response

1. Multiple Sclerosis (MS): The patient's MRI findings of multiple non-enhancing T2 hyperintense lesions in the supratentorial white matter, periventricular white matter, and corpus callosum, along with the presence of oligoclonal bands in the CSF, are suggestive of MS. However, the absence of clinical signs of optic neuritis, spinal cord symptoms, and the presence of gaze-evoked nystagmus make MS less likely.

2. Sneddon Syndrome: This condition, characterized by livedo reticularis and cerebrovascular disease, could explain the patient's cognitive decline and MRI findings. However, the absence of livedo reticularis and the presence of gaze-evoked nystagmus do not support this diagnosis.

3. Susac Syndrome: This is a rare disorder characterized by the triad of encephalopathy, branch retinal artery occlusion, and hearing loss, which matches some of the patient's symptoms and findings. The MRI findings of T2 hyperintense lesions in the corpus callosum are also seen in Susac syndrome. However, the patient's age (it usually affects people aged 20-40) and the absence of typical "snowball" MRI lesions make this diagnosis less likely.

4. Cerebral Amyloid Angiopathy (CAA): This condition can cause cognitive decline and cerebral microbleeds, which could explain the patient's symptoms and MRI findings. However, the patient's relatively young age and the absence of lobar, cortical, or cortico-subcortical cerebral microbleeds make CAA less likely.

5. Vasculitis: This could cause cognitive decline and multiple cerebral lesions. However, the absence of systemic symptoms such as fever, malaise, weight loss, and anemia, and the normal cell count in the CSF make this diagnosis less likely.
    a. Primary CNS Vasculitis: This condition could explain the patient's cognitive decline and MRI findings. However, the absence of systemic symptoms and the normal cell count in the CSF make this diagnosis less likely. Primary CNS vasculitis typically presents with headaches,

cognitive impairment, and focal neurologic deficits, and MRI often shows multiple infarcts or hemorrhages.

b. Systemic Vasculitis (e.g., Granulomatosis with Polyangiitis, Lupus): These conditions could also cause neurologic symptoms and MRI changes due to inflammation of the blood vessels in the brain. However, these conditions usually present with systemic symptoms and signs such as fever, weight loss, skin rash, joint pain, and kidney disease, which are not reported in this patient.

6. Infectious or Post-Infectious Encephalitis: Given the patient's recent upper respiratory symptoms, an infectious or post-infectious cause could be considered.
    a. Viral Encephalitis (e.g., Herpes Simplex Virus, Varicella-Zoster Virus, Enteroviruses): These viruses can cause encephalitis with symptoms of cognitive decline and MRI changes. However, these conditions usually present with fever and CSF pleocytosis, which are not reported in this patient.
    b. Post-Infectious or Autoimmune Encephalitis (e.g., Anti-NMDA Receptor Encephalitis, Anti-LGI1 Encephalitis): These conditions can cause subacute cognitive decline and MRI changes due to an autoimmune response triggered by a previous infection. However, these conditions often present with other neurologic symptoms such as seizures, movement disorders, and psychiatric symptoms, which are not reported in this patient.

7. Toxic or Metabolic Encephalopathy: The patient's positive toxicology screen for cannabinoids and amphetamines raises the possibility of a toxic or metabolic cause. However, the patient's cognitive decline persisted despite removal from the potential toxic environment, making this less likely.

8. Leukodystrophies: These are rare genetic disorders that affect the white matter of the brain. The MRI findings could be suggestive of a leukodystrophy. However, these conditions usually present in childhood or adolescence, making this diagnosis less likely in a 44-year-old patient.

9. Neoplastic or Paraneoplastic Syndromes: These could cause cognitive decline and multiple cerebral lesions. However, the absence of systemic symptoms such as weight loss, night sweats, and the absence of enhancement of the lesions on MRI make this diagnosis less likely.

Score: 0

- Neoplastic and leukodystrophies are deemed aberrant.

<u>Case 4</u>

Question 4.2 – AI’s response

The patient's clinical presentation, laboratory findings, and imaging results suggest a diagnosis of Paraneoplastic Neuropathy.

Here's why:

The patient's nerve conduction studies and electromyography results show evidence of a peripheral neuropathy with conduction block in the forearm of the bilateral ulnar and left median nerves, mildly slowed conduction velocity of the left median and ulnar motor nerves, and widespread active denervation in upper and lower extremities on the left side.

The patient's CSF analysis revealed an elevated cell count and protein level, which can be seen in paraneoplastic syndromes.

The patient's recent weight loss and mildly elevated CSF cell count prompted further workup for systemic disease. The contrast-enhanced CT of the chest, abdomen, and pelvis showed lymphadenopathy in the mediastinum, upper abdomen, and hilar regions, which could suggest an underlying malignancy causing a paraneoplastic syndrome.

The patient's mildly reduced vitamin B12 level could potentially contribute to the neuropathy, but the normal methylmalonic acid level makes a B12 deficiency less likely as the primary cause.

In conclusion, while the patient's presentation is complex and could potentially be explained by several different conditions, the combination of peripheral neuropathy, elevated CSF cell count and protein level, and systemic signs of malignancy suggest a paraneoplastic neuropathy. Further workup, including potentially a biopsy of the lymph nodes, would be necessary to confirm the diagnosis and identify the underlying malignancy.

Score: 0

- only neurosarcoidosis was deemed correct.
